# Initial Insights Into the Genetic Epidemiology of SARS-CoV-2 Isolates From Kerala Suggest Local Spread From Limited Introductions

**DOI:** 10.3389/fgene.2021.630542

**Published:** 2021-03-17

**Authors:** Chandni Radhakrishnan, Mohit Kumar Divakar, Abhinav Jain, Prasanth Viswanathan, Rahul C. Bhoyar, Bani Jolly, Mohamed Imran, Disha Sharma, Mercy Rophina, Gyan Ranjan, Paras Sehgal, Beena Philomina Jose, Rajendran Vadukkoot Raman, Thulaseedharan Nallaveettil Kesavan, Kalpana George, Sheela Mathew, Jayesh Kumar Poovullathil, Sajeeth Kumar Keeriyatt Govindan, Priyanka Raveendranadhan Nair, Shameer Vadekkandiyil, Vineeth Gladson, Midhun Mohan, Fairoz Cheriyalingal Parambath, Mohit Mangla, Afra Shamnath, Indian CoV2 Genomics & Genetic Epidemiology (IndiCovGEN) Consortium, Sridhar Sivasubbu, Vinod Scaria

**Affiliations:** ^1^Government Medical College, Kozhikode, India; ^2^Council of Scientific and Industrial Research (CSIR)-Institute of Genomics and Integrative Biology, New Delhi, India; ^3^Academy of Scientific and Innovative Research, Ghaziabad, India

**Keywords:** COVID-19, COVIDSeq, Kerala, genetic epidemiology, variants

## Abstract

Coronavirus disease 2019 (COVID-19) rapidly spread from a city in China to almost every country in the world, affecting millions of individuals. The rapid increase in the COVID-19 cases in the state of Kerala in India has necessitated the understanding of SARS-CoV-2 genetic epidemiology. We sequenced 200 samples from patients in Kerala using COVIDSeq protocol amplicon-based sequencing. The analysis identified 166 high-quality single-nucleotide variants encompassing four novel variants and 89 new variants in the Indian isolated SARS-CoV-2. Phylogenetic and haplotype analysis revealed that the virus was dominated by three distinct introductions followed by local spread suggesting recent outbreaks and that it belongs to the A2a clade. Further analysis of the functional variants revealed that two variants in the *S* gene associated with increased infectivity and five variants mapped in primer binding sites affect the efficacy of RT-PCR. To the best of our knowledge, this is the first and most comprehensive report of SARS-CoV-2 genetic epidemiology from Kerala.

## Introduction

The coronavirus disease 2019 (COVID-19) pandemic has seen a widespread application of genomic approaches to understand the epidemiology and evolution of SARS-CoV-2. The accelerated efforts to sequence genomes of clinical isolates of SARS-CoV-2 from across the world picked up pace following the initial genome sequencing of the virus from a patient in Wuhan, the epicenter for the pandemic (Wu et al., 2020). As the virus evolves through the accumulation of mutations, it has split into major lineages with strong geographical affinities (Li et al., 2020b). The availability of the genome sequences in the public domain has provided a unique view of the introduction, evolution, and dynamics of SARS-CoV-2 in different parts of the world (Shu and McCauley, 2017; Rito et al., 2020).

A number of approaches have emerged for rapid and scalable sequencing of SARS-CoV-2 from clinical isolates. This includes direct shotgun approaches, targeted amplicon-based, and targeted capture-based approaches (Meredith et al., 2020; Yángüez et al., 2020). Sequencing based approaches provide a unique opportunity for high fidelity of detection and for understanding the genetic epidemiology of SARS-CoV-2 (Bhoyar et al., 2020). Additionally, the genetic variants could offer insights into the mutational spectrum, evolution, infectivity, and attenuation of the virus (Muth et al., 2018; Korber et al., 2020). Additional analyses on genomic variants have also provided useful insights into the efficacy of primer/probe-based diagnostic assays as well as immune epitopes and resistance to antisera (Grifoni et al., 2020; Jain et al., 2020).

Kerala is a unique state in India with a population of 35 million people and extensively connected with the global populations through over 1.6 million expatriates. The state is in a distinct position, affected by local as well as global epidemics. In fact, the first identified case of COVID-19 in India was from Kerala. The patient had traveled from Wuhan, China (Yadav et al., 2020), the genomic identity of which mapped to the Nextstrain clade B of SARS-CoV-2 (Somasundaram et al., 2020). Further introductions into the state during the later days of the pandemic through international and regional travel could have contributed to the spread of the epidemic in the state. Since the beginning of the pandemic in the country, India has seen a shift in the prevalence of different SARS-CoV-2 variants in different states. The early months of the pandemic (March and April) was dominated by the clade I/A3i, a distinct phylogenetic cluster reported from the genomes in India, while by late April, a shift in clade prevalence was observed as most states showed an increased representation of the Nextstrain clade A2a (Banu et al., 2020). While a number of studies on the genetic epidemiology of SARS-CoV-2 from different states in India have emerged (Banu et al., 2020; Somasundaram et al., 2020), there has been a paucity of genomic data for SARS-CoV-2 from Kerala needed to assess the genetic epidemiology of COVID-19 and the prevalence of different lineages of the virus under circulation in the state.

We intended to fulfill the gap in knowledge on the identity of the circulating genetic lineages/clades contributing to the epidemic in Kerala. To this end, we employed a high-throughput sequencing-based approach for the SARS-CoV-2 genetic epidemiology. To the best of our knowledge, this is the first comprehensive overview of the genetic architecture of SARS-CoV-2 isolates from the state of Kerala.

## Methods

### Samples and RNA Isolation

The study is in compliance with relevant laws and institutional guidelines and in accordance with the ethical standards of the Declaration of Helsinki and approved by the institutional Human Ethics Committee approved the project (GMC KKD/RP2020/IEC438). The volunteers were well-informed and had written consent about participation and knowledge about the study. RNA samples were isolated from nasopharyngeal/oropharyngeal swabs of patients with informed written consent presenting to Government Medical College, Kozhikode, Kerala. Samples included in this study were collected between 19/07/2020 and 07/08/2020. RNA extraction was done using MagMax Viral/Pathogen Nucleic Acid Isolation kit in Thermo Scientific KingFisher Flex automated extraction system according to the manufacturer's instructions. All the RNA samples were transferred within 72 h of collection at a cold temperature (2–8°C) and were stored at −80°C until further processing.

### Sequencing and Data Processing

Sequencing was performed using the COVIDSeq amplicon-based next generation sequencing test (Illumina, Inc.) as reported previously (Bhoyar et al., 2020). Briefly, this protocol involved 2019-nCoV primers designed to detect RNA from the SARS-CoV-2 virus followed by the multiplex amplicon sequencing on the Illumina NovaSeq platform. The base calls generated in the binary base call (BCL) format were demultiplexed to FASTQ reads using bcl2fastq (v2.20). For reference-based assembly, we followed a previously defined protocol from Poojary et al. (2020). As per the protocol, the quality control of FASTQ reads was performed using Trimmomatic (v0.39) at a Phred score of Q30 (Bolger et al., 2014) with adapter trimming. These reads were further aligned to the severe acute respiratory syndrome 2 (SARS-CoV-2) reference genome (NC_045512.2) using HISAT2-2.1 (Kim et al., 2015; Wu et al., 2020). The human reads were removed using SaMtools (v1.10) (Li et al., 2009). The samples with coverage >99% and <5% unassigned nucleotides underwent variant calling and consensus sequences generation using VarScan (v2.4.4) (Koboldt et al., 2009) and SaMtools (v1.10) (Li et al., 2009), bcftools (v1.10.2), and seqtk (v 1.3-r114) (Shen et al., 2016), respectively.

### Variant Annotation and Comparison With Existing Datasets

Variants were annotated using ANNOVAR (Wang et al., 2010), employing a range of custom annotation datasets and tables. All the variants identified were systematically compared with a compendium of other Indian and global variants. A total of 93,995 complete SARS-CoV-2 genomes deposited in the Global Initiative on Sharing All Influenza Data (GISAID) database till September 1, 2020, were used for comparative analysis (Supplementary Table 1). Viral genomes with a pairwise alignment ≥99% and gaps <1% with the reference genome (NC_045512.2) were considered for further variant calling using SNP sites (Page et al., 2016). Genetic variants compiled from a total of 1,855 high-quality genomes from India and 32,286 global genomes were considered for analysis.

### Phylogenetic Analysis

Phylogenetic analysis was performed according to the pipeline provided by Nextstrain (Hadfield et al., 2018). The dataset of 2,476 complete SARS-CoV-2 genomes deposited in the GISAID database from India was used for the analysis (Supplementary Table 2), along with 113 genomes from the current study, which have 99% coverage and at least 98% pairwise alignment with the reference genome (NC_045512.2). Genomes having more than 5% Ns or missing dates of sample collection were excluded from the analysis. The phylogenetic tree was constructed and refined to a molecular clock phylogeny using the Augur framework provided by Nextstrain and was visualized using Auspice. The Phylogenetic Assignment of Named Global Outbreak LINeages (PANGOLIN, version 2020-07-20) package was used to assign lineages to the genomes from this study (Rambaut et al., 2020). The lineages were visualized and annotated on the phylogenetic tree using iToL (Letunic and Bork, 2019).

### Haplotype Analysis

For haplotype analysis, the genomes were aligned to the SARS-CoV-2 (NC_045512.2) reference genome using MAFFT (Katoh and Toh, 2008) and problematic genomic loci (low coverage, high sequencing error rate, and hypermutable and homoplasic sites) were masked from the alignment (De Maio et al., 2020). The aligned sequences were imported into the DNA Sequence Polymorphism tool (DnaSP v6.12.03) (Rozas et al., 2017) to generate haplotypes. A TCS haplotype network (Clement et al., 2000) for the genomes was constructed using the Population Analysis with Reticulate Trees software (POPART v 1.7) (Leigh and Bryant, 2015). Times to the most recent common ancestor (tMRCA) for the haplogroups were computed following the Bayesian Markov chain Monte Carlo (MCMC) method using BEAST v1.10.4 (Suchard et al., 2018) following a previously defined protocol for phylodynamic analysis of SARS-CoV-2 genomes (Bedford, 2020, Rambaut, 2020). Tip dates were specified using sample collection dates of the individual genome sequences and samples having ambiguous or missing dates of collection were excluded from the analysis. The analysis was performed using a coalescent growth rate model along with a strict molecular clock and the HKY+Γ substitution model with gamma-distributed rate variation (gamma categories = 4). MCMC was run for 50 million steps. The output was analyzed in Tracer v1.7.1 (Rambaut et al., 2018) and burn-in was adjusted to attain an appropriate effective sample size (ESS).

### Functional SARS-CoV-2 Variants and Selection Pressure Analysis

Further, we have evaluated the SARS-CoV-2 variants based on their functional relevance. We curated a comprehensive compendium of SARS-CoV-2 variants of functional relevance that are associated with increased infectivity and attenuation of SARS-CoV-2 from literature. The variants were systematically annotated and mapped to the reference genome coordinates and their respective amino acid changes. This variant compendium encompassed about 337 variants curated from 35 publications. The variants in this study were compared with the genomic variants generated using bespoke scripts. We analyzed selection pressure acting on codons of the genes ORF1a and ORF1b and the genes that encode different structural proteins—Spike (S), Envelope (E), Membrane (M), and Nucleocapsid (N) proteins for the dataset of Indian SARS-CoV-2 genomes used in the study. Ratios of non-synonymous (dN) and synonymous (dS) substitution rates were calculated using HyPhy (Pond et al., 2005). A total of three different codon-based estimations were used to detect positive selection, i.e., mixed effects model of evolution (MEME), single likelihood ancestor counting (SLAC), and fixed effects likelihood (FEL). Only amino acid positions that were found to be under positive selection by all three methods were considered.

### Variant Effect on RT-PCR Efficacy

We were also interested to evaluate the effect of SARS-CoV-2 variants on the efficacy of RT-PCR detection. We took a compiled list of 132 primer/probe sequences widely used in the molecular detection of SARS-CoV-2 around the globe (Jain et al., 2020). In our analysis, we mapped the Kerala isolate SARS-CoV-2 genetic variants to the 132 primer/probe sequence and calculated the melting temperature (Tm) of the mutant with the wild-type sequence. The length of primers in the curated list is >13 nucleotides, so we applied this formula for calculating melting temperature Tm = 64.9 + 41^*^(yG + zC – 16.4)/(wA + xT + yG + zC) where w, x, y, and z are the number of A, T, G, and C nucleotides, respectively (Wallace et al., 1979).

## Results

### Sequencing and Data Processing

**Figure 1:**
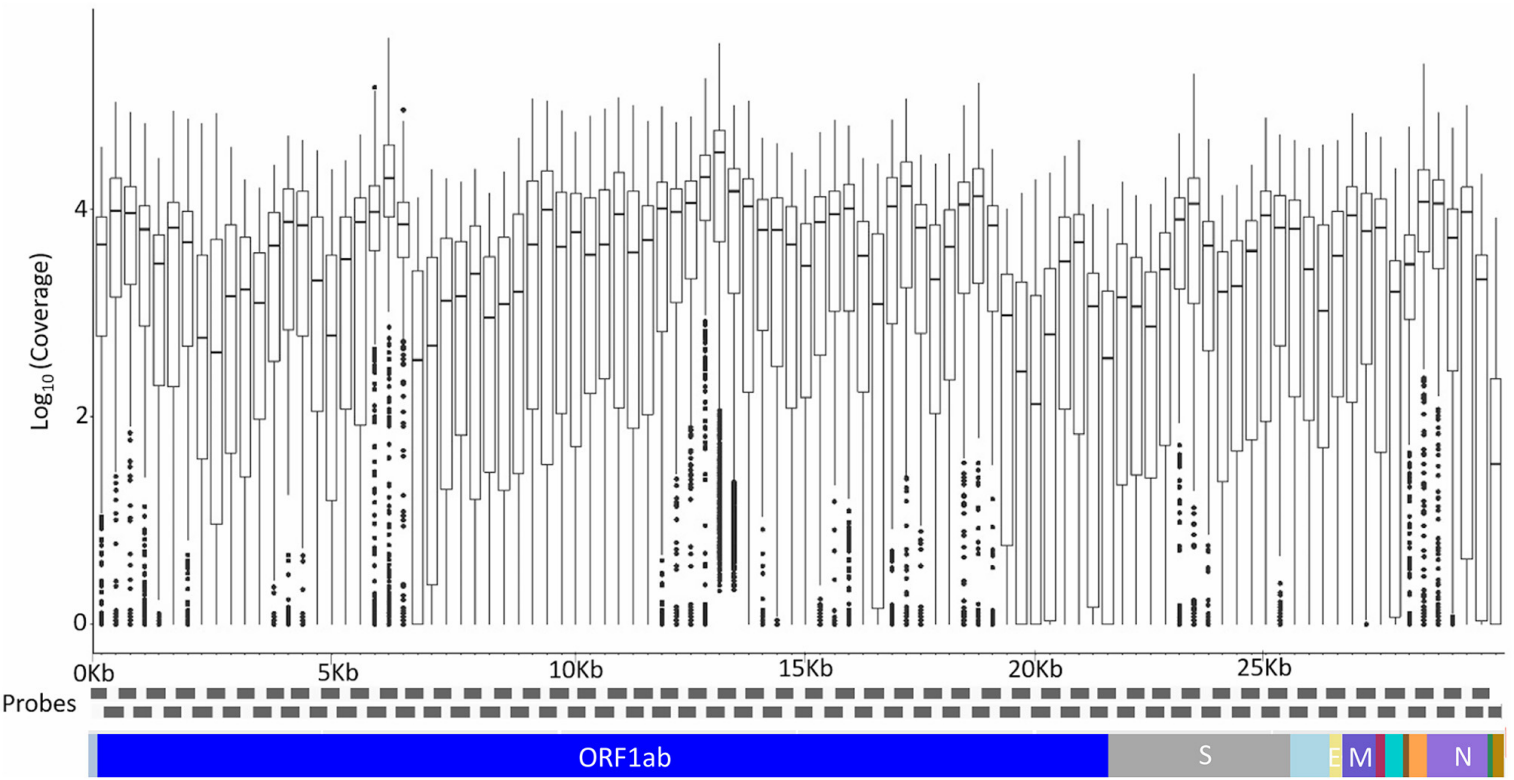
The mean coverage of the SARS-CoV-2 genomes across the amplicons of the COVIDSeq amplicon-based sequencing.

A total of 200 Kerala isolates of SARS-CoV-2 were processed for genome sequencing. The genomes were sequenced using amplicon-based COVIDSeq protocol (Bhoyar et al., 2020) and generated ~8.1 million raw reads per sample. The reads were subjected to quality control and resulted in ~7.5 million reads per sample, of which around 6.4 million reads per sample aligned to the SARS-CoV-2 reference genome (NC_045512.2). The reads had a mapping percentage of 84.93% and 7,755× mean coverage. The data have been summarized in Supplementary Table 3, and the mean coverage of the sample across the amplicons has been represented in Figure 1.

### Variant Annotation and Comparison With Existing Datasets

**Figure 2:**
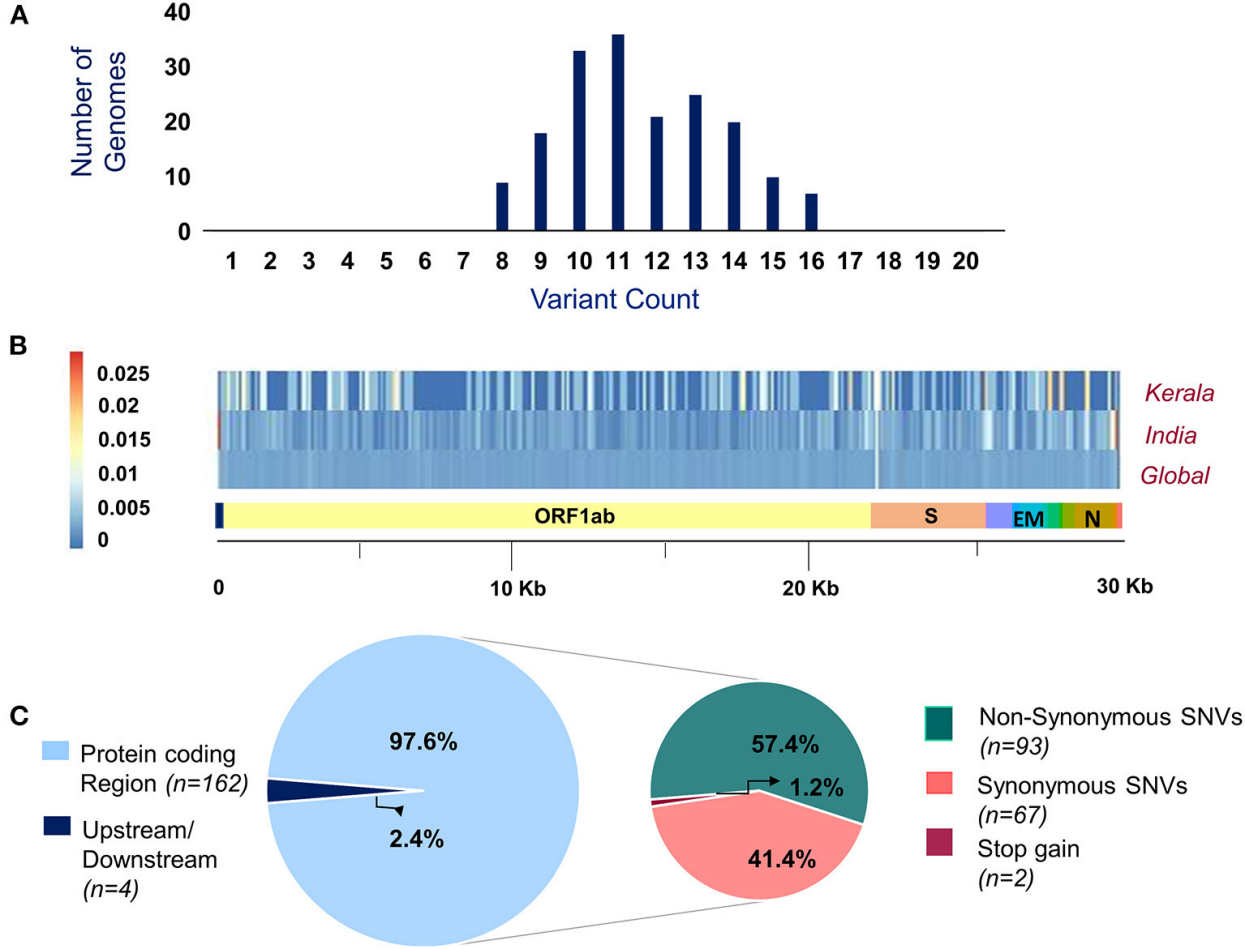
**(A)** Distribution of variants across genomes used in the study. **(B)** Comparison of the proportion of the variants represented with their allele frequency across the SARS-CoV-2 genome in datasets includes Kerala (present study), India, and global. **(C)** Distribution of the genetic context of variants and their functional classification.

Of the 200 SARS-CoV-2 isolates sequenced, a total of 179 samples had >99% coverage and <5% unassigned nucleotides across the genome. These samples were further processed for variant calling and consensus generation. Our analysis identified a total of 195 unique variants, with a median variant count of 12 per sample. Variant quality has been ensured with the average variation percentage across genomes ≥50. Of the total 195 unique variants, 166 were categorized as high-quality variants (Supplementary Table 4). The distribution of variants across the SARS-CoV-2 genomes used in the study was analyzed. Also, the proportional distribution of variants for every 100 bps across the genome was calculated and compared among various datasets and is presented in Figure 2. Out of the 166 high-quality unique variants, four variants were novel (Supplementary Table 5) and 89 new variants (2.61%) were added to the Indian repertoire of genetic variants compiled in Supplementary Table 6. The overlap in the variants between the present study of Kerala, other Indian datasets, and global datasets is summarized in Supplementary Figure 1. Out of the four novel variants, one variant in the S gene, 25281G>A, was a personal variant and was not shared by any other isolate. The remaining three novel variants were shared variants and were present in different genes (Orf1b, Orf7a, and S).

### Genomic Context and Classification of the Variants

Of the total 166 high-quality unique variants, 162 variants were located in the protein-coding regions while four variants mapped to either downstream or upstream regions. Of the total variants in protein-coding regions, 93 variants were non-synonymous, 67 were synonymous, and two variants resulted in stopgain mutation as presented in Figure 2. These two stopgain variants were found in ORF3a (26113:G>T) and ORF8 (28028:G>A) genes and were present in one genome isolate each.

### Phylogenetic Analysis

**Figure 3:**
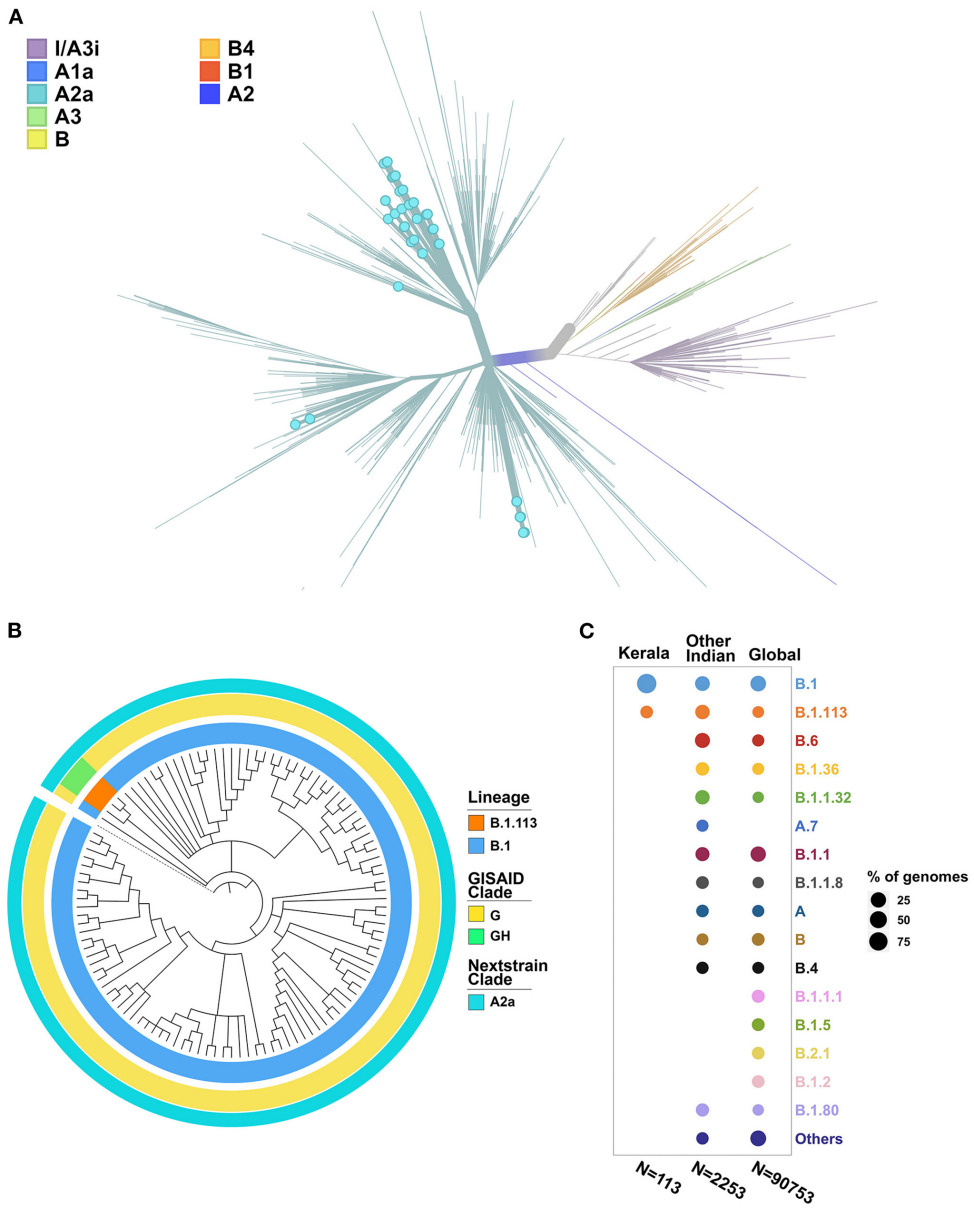
**(A)** Phylogenetic map of the 113 genomes sequenced from Kerala (highlighted by blue dots) with respect to the other genomes from India. **(B)** Distribution of the clades and lineages in Kerala. All genomes clustered under the clade A2a (GISAID clade G and GH) while the dominant lineage was B.1. **(C)** Lineage distribution in Kerala compared to the distribution across India and global populations.

The phylogenetic tree was constructed using the genome Wuhan/WH01 (EPI_ISL_406798) as root and 2,366 genomes from India that met the inclusion criteria (Ns < 5%, no missing/ambiguous date of sample collection) including 113 genomes sequenced in this study. All 113 genomes from this study were found to cluster under the globally predominant Nextstrain clade A2a (GISAID clade G and GH). In contrast, one of the previous genomes available from Kerala (EPI_ISL_413523, submitted by National Institute of Virology, Pune, India), which is also one of the first SARS-CoV-2 genomes sequenced in India, belongs to the Nextstrain clade B (Yadav et al., 2020). The dominant lineage assigned by PANGOLIN for the 113 genomes was found to be B.1 (*n* = 110), while three genomes were assigned the lineage B.1.113. The phylogenetic map of the dataset of Indian genomes and the distribution of lineages in the 113 genomes from Kerala are summarized in Figure 3.

### Haplotype Analysis

**Figure 4:**
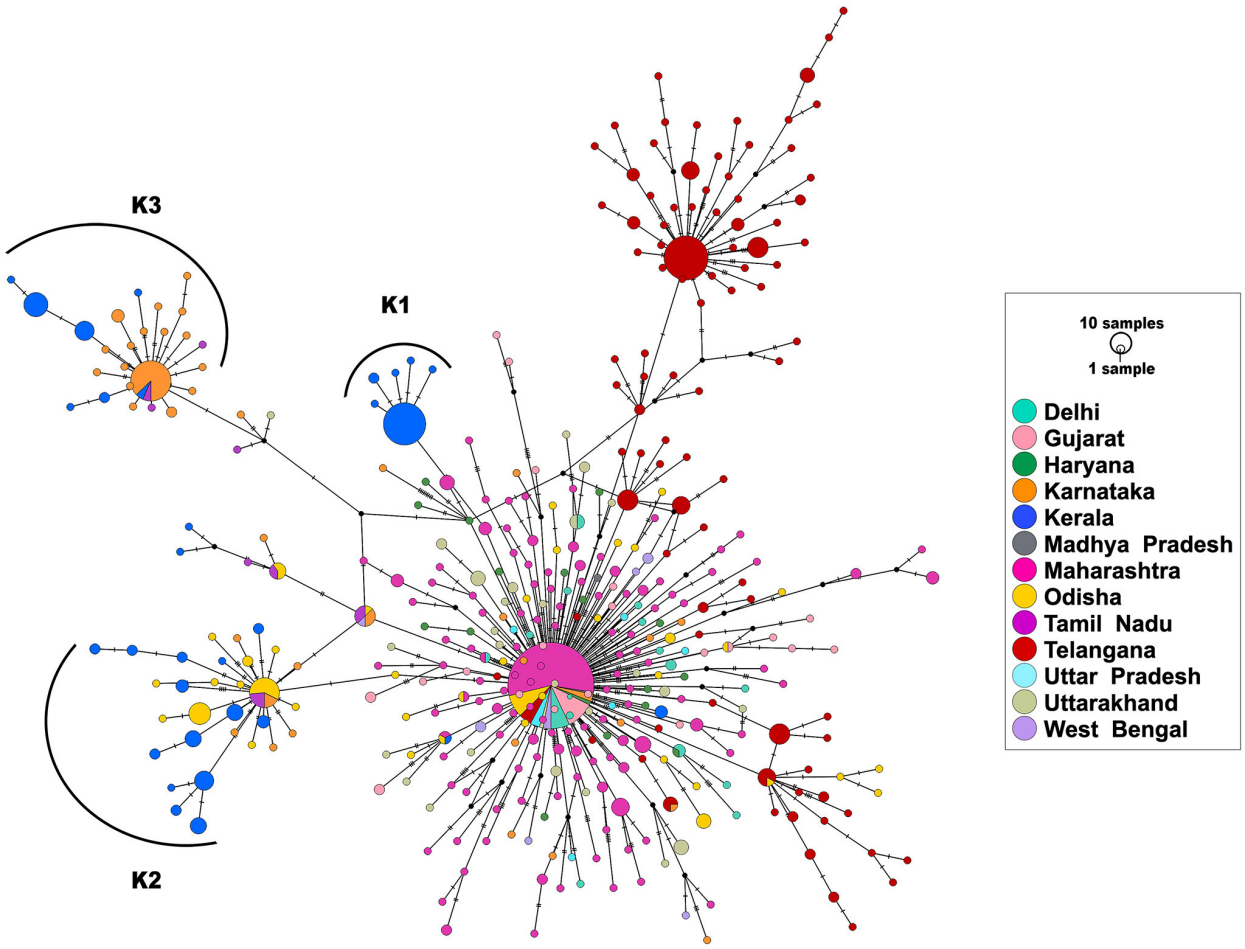
Haplotype network of 850 genomes of Indian isolates of SARS-CoV-2 belonging to the A2a clade. The three major haplogroups encompassing the genomes from Kerala are designated as K1, K2, and K3.

Haplotype analysis was done using a dataset of 850 SARS-CoV-2 genomes from India (including 113 genomes from Kerala) that fell under clade A2a in the phylogenetic tree and clustered close to the 113 genomes from Kerala. Among the 850 genomes, there were 592 variable sites and 400 unique haplotypes (Supplementary Table 7). Mutations unique to the three haplotypes, their frequency of occurrence in the 113 genomes from Kerala, and information about the first detected genome having the mutation in Indian and global datasets are given in Supplementary Table 8. The haplotype network as generated by POPART shows that a few haplogroups contributed to a majority of the isolates. Three major haplogroups contributed to 94.6% of the isolates from Kerala. The major haplogroup (K1) encompassed 40 genomes from Kerala (35.4%). The network suggests that the cluster K1 had a potential ancestor from the state of Maharashtra before possible introduction and dissemination in Kerala. A variant 16726C>T was observed to be common between the 40 genomes as well as the three genomes from Maharashtra belonging to the ancestral haplotype. The K1 cluster also included four genomes from Kerala that were found to be in a polytomy in the phylogenetic tree. Close follow-up of the cases suggests a local outbreak that contributed to the polytomy. The second haplogroup (K2) encompasses 42 genomes (37.1%) from Kerala and shares 27 genomes from Odisha. In addition, five genomes from Kerala in this group also constitute a polytomy. The third group (K3) encompasses 25 genomes (22.1%) from Kerala and shares 46 genomes from Karnataka. Figure 4 summarizes the haplotype network of the A2a clade genomes.

To understand the times of introduction, tMRCA was computed for the three distinct haplogroups. The median tMRCA were 14 July 2020 (95% highest posterior density interval [HPD] 11 May−22 July), 20 March 2020 (95% HPD 12 Feb−16 May), and 6 April 2020 (95% HPD 3 March−27 May) for the three major haplogroups K1, K2, and K3, respectively. Taken together, the analysis suggests that the majority of the SARS-COV-2 isolates are outcomes of limited introductions early in the epidemic followed by local circulation.

### Functional Consequences and Selection Pressure of the Variants

Annotating the variants for their functional consequences using custom annotation datasets revealed a total of 42 genetic variants that were predicted as deleterious by SIFT (Ng and Henikoff, 2003). The filtered variants were found to span 13 unique protein domains as per UNIPROT (The UniProt Consortium, 2017) annotations. We found 15 and 120 genetic variants that mapped back to potential B and T cell epitopes from the Immune Epitope Database (IEDB) (Vita et al., 2019), respectively. In addition, five variants were found to span predicted error-prone sites including sequencing error sites, homoplasic positions, and hypermutable sites. Functional annotation details of all the filtered variants are summarized in Supplementary Table 9. Detection of positive pressure for the codons of ORF1a, ORF1b, S, E, M, and N genes was performed using MEME, SLAC, and FEL methods. A total of 2,366 genomes from India including 113 genomes sequenced in this study were used for the analysis. We have identified 19 amino acid sites that were under positive selection by all three methods, 11 of which are in ORF1a, six in ORF1b, and two in S gene (Supplementary Table 10). Five amino acid positions under positive selection also corresponded to variant sites that mapped to potential CD4 and CD8 epitopes (Supplementary Table 10).

### Variants in Diagnostic Primer/Probe Binding Sites in the Genome

We also explored whether the variants mapped to the RT-PCR primers and probes sites. On mapping the genetic variants with the curated primers and probes, we found five unique variants at five unique primer or probes binding sites. A total of four unique variants had allele frequency > 1% at four unique primer binding sites. The majority of the variants, i.e., four, lies in the primer binding sites in *ORF1b, S, E*, and *N* with an allele frequency of 0.559, 4.469, 1.117, and 3.352% in the Kerala isolate genomes, respectively. While a variant 28899:G>T mapped to the 2019-nCoV-NFP, which is a part of China Centers for Disease Control and Prevention (CDC) primer set with a frequency of 1.117%, the Tm differed in the mutated sequence by the unit of ±2 in comparison to the wild-type sequence. The variant 22444C>T having a frequency of 4.469% in the Kerala genome isolates corresponds to the codon position 294 in S gene, which was found to be under positive selection (Supplementary Table 10). Summary of novel variants and diagnostic primer/probe spanning variants are compiled in Supplementary Tables 5, 11, respectively. Details on the read count and depth of coverage of these variants are systematically documented in Supplementary Tables 12A,B.

### Variants Associated With Potential Increased Infectivity or Attenuation of the Virus in Experimental Settings

With the view of identifying potential functionally relevant variants, we overlapped the variants obtained from the present study with a manually curated compilation of functionally relevant SARS-CoV-2 variants. Our analysis identified two variants in the S gene, which were reported to be associated with increased infectivity. L5F, a variation co-occurring with D614G, was earlier demonstrated to possess increased infectivity (Korber et al., 2020; Li et al., 2020a; Plante et al., 2020) using cell line studies. In our study, 23403A>G (D614G) and 21575C>T (L5F) mutations were observed at frequencies of 99.44 and 15.64%, respectively, in the genomes. The combination of these variations was found to occur at a higher frequency in genomes from Kerala.

## Discussion

Within a small time frame, SARS-CoV-2 has spread from Wuhan to countries across the world affecting over 26 million individuals.^1^ The virus evolves by accumulating variants at an almost constant rate of 1.19–1.31 × 10^−3^ base substitutions per site per year (Li et al., 2020b) and therefore leaves the mutational fingerprint, which is widely used for tracing the spread of the virus (Rafiul Islam et al., 2020). The availability of high-throughput sequencing approaches has enabled researchers to sequence genomes as the pandemic progressed in their respective countries. A number of methods have been adopted for rapid high-throughput sequencing of SARS-CoV-2 including shotgun sequencing (Meredith et al., 2020), PCR amplicon, and hybridization/capture-based enrichment and sequencing (Bhoyar et al., 2020; Yángüez et al., 2020).

Genome sequencing of SARS-CoV-2 in various countries [COVID-19 Genomics UK (COG-UK) consortiumcontact@cogconsortium.uk, 2020] has led to insights into the temporal and geographical spread of the virus (Alm et al., 2020), introductions, and spread of the virus through travelers (Oude Munnink et al., 2020), local transmission, and dynamics (Lu et al., 2020), investigating the origin of outbreaks (Huang et al., 2020), just to name a few. By virtue of its connectivity to major cities through its expatriate population, trade and tourism is uniquely poised in this pandemic. It is not surprising therefore that the first case of COVID-19 in India, early in the pandemic, was reported from Kerala (Yadav et al., 2020). The genome of the isolate suggested that it originated from China (Yadav et al., 2020). The following months have seen the number of cases increase to over 80,000 in the state with a paucity of information on the origin, spread, and dynamics of the virus.^2^

In this present study, we performed sequencing and analysis of SARS-CoV-2 isolates from Kerala, which revealed unique patterns of the transmission. These genomes are clustered into a monophyletic group mapping to the A2a clade. The A2a clade is also marked by the D614G variant, which is suggested to confer higher infectivity, efficient replication and transmission in *in vitro* and *in vivo* (Baric, 2020; Hou et al., 2020; Hu et al., 2020; Zhang et al., 2020) and is therefore thought to have emerged globally as the predominant clade (Korber et al., 2020) from a probable origin in Europe (Rito et al., 2020). Haplotype analysis suggests that three major haplogroups with distinct ancestry groups suggest that the introductions were from inter-state travel. The prevalent haplotypes were not found in any of the global genomes, supporting this observation. Strict travel restrictions, particularly air travel, have previously been shown to lower the spread of the disease (Rito et al., 2020) and the phylogeographic analysis in this study suggests that focused testing, tracing, and quarantine of expatriates and international travelers implemented during the epidemic would have been effective in curbing the spread from international travelers. The genome clusters also suggested polytomies, suggesting a recent outbreak (Banu et al., 2020). Close follow-up of the cluster members confirmed the potential source of the outbreak, suggesting that genetic epidemiology could be used in conjunction with case follow-ups to uncover potential outbreaks and possibly connect outbreaks that are apparently not related.

This study uncovered a total of four novel genetic variants and 89 variants that were identified only in Kerala and not in the rest of India. The genome sequences could also uncover insights into the variants of functional relevance. One of the variants of significance is a stopgain variant (28028:G>A) in the ORF8 gene. Variants including deletions in ORF8 have been suggested to attenuate the virus (Gaurav et al., 2020; Young et al., 2020). Similar variants have also been identified in other related viruses like the SARS-CoV and MERS-CoV (Lamers et al., 2016; Muth et al., 2018). A variant 21575C>T (L5F) in the *S* gene associated with increased infectivity of the virus (Li et al., 2020a) was present in 15.64% of the genomes sequenced. Following recent reports that suggest that variants in the primer/probe binding sites could impact the efficiency of RT-PCR assays (Jain et al., 2020; Khan and Cheung, 2020), we explored whether any of the variants in the present study mapped to the primer/probe binding sites. We identified five unique variants in five unique binding sites. The maximum number of variants were the primer set published by Won et al. (2020) spanning multiple genes, apart from the 2019-nCoV-NFP GGGGAACTTCTCCTGCTAGAAT binding sites in the N gene (World Health Organization, 2020). The latter is part of the China Centers for Disease Control and Prevention (CDC) protocol with variants in 1.117% in genomes from Kerala. We have earlier reported variants in this primer site in 39.5% of the genomes from India (Jain et al., 2020) and 18.8% (Khan and Cheung, 2020) of global genomes. This information would be potentially valuable for laboratories in selecting reagents for screening and diagnosis.

The study has two caveats; the first is that the samples were collected from a single major tertiary care center in North Kerala. However, the center caters to a large population and region and has close proximity to an international airport. Secondly, the sampling was limited to a short period of time, thus enabling only a cross-sectional view of the epidemic and precluding an accurate and temporal view of the dynamics of the epidemic in the state. Nevertheless, this provides a unique opportunity to create a snapshot of the epidemic in time and space. Notwithstanding the limitations, this is the first and most comprehensive overview of the genetic epidemiology of SARS-CoV-2 in the state of Kerala. While providing insights into the epidemiology of the epidemic, the study also enabled tracing outbreaks, thereby highlighting the utility of genome sequencing as an adjunct to high-throughput screening and testing. It has not escaped our mind that scalable technologies that can combine both the approaches (Bhoyar et al., 2020) could potentially find a place in understanding epidemics better.

## Data Availability Statement

The datasets presented in this study can be found in online repositories. The names of the repository/repositories and accession number(s) can be found at: https://www.ncbi.nlm.nih.gov/, PRJNA662193.

## Ethics Statement

The studies involving human participants were reviewed and approved by Institutional Human Ethics Committee approved the project (GMC KKD/RP2020/IEC438). The patients/participants provided their written informed consent to participate in this study.

## Author Contributions

CR: methodology, validation, investigation, resources, and writing—original draft. MD, RB, MI, GR, and PS: validation, investigation, resources, and writing—original draft. AJ, BJ, DS, and MR: software, formal analysis, data curation, and writing—original draft. PV, BPJ, RR, TK, KG, SM, JP, SK, PN, SV, VG, MMo, and FP: validation and investigation. MMa and AS: formal analysis and data curation. SS and VS: conceptualization, methodology, supervision, project administration, and writing—original draft. All authors contributed to the article and approved the submitted version.

## Conflict of Interest

The authors declare that the research was conducted in the absence of any commercial or financial relationships that could be construed as a potential conflict of interest.

## Footnotes

^1^Summary table of available protocols in this document. Available online at: https://www.who.int/docs/default-source/coronaviruse/whoinhouseassays.pdf?sfvrsn=de3a76aa_2 (accessed September 16, 2020).

^2^Kerala: COVID-19 Battle. Available online at: https://dashboard.kerala.gov.in/ (accessed September 16, 2020).

## Supplementary Material

Supplementary Figure 1 Distribution of genetic variants across datasets.

Supplementary Table 1 GISAID acknowledgment table for global genomes used in the study.

Supplementary Table 2 GISAID acknowledgment table for genomes from India considered for phylogenetic analysis.

Supplementary Table 3 Data summary of the samples sequenced by COVIDSeq Protocol and processed using a custom pipeline.

Supplementary Table 4 Summary of quality details of the variants identified in the study.

Supplementary Table 5 Summary of novel genetic variants and their frequencies in the dataset.

Supplementary Table 6 Compilation of genetic variants identified for the first time in Indian genomes.

Supplementary Table 7 Unique haplotypes for the dataset of 850 genomes belonging to clade A2a.

Supplementary Table 8 Mutations unique to the three haplogroups and their frequency in Kerala.

Supplementary Table 9 Summary of functional annotation of unique genetic variants identified in the study.

Supplementary Table 10 Amino-acid sites under positive selection by MEME, SLAC and FEL methods.

Supplementary Table 11 Summary of variants mapping to diagnostic RT-PCR primers or probes.

Supplementary Table 12A Tabulation of read alignment statistics for variants spanning Primer/Probe sites.

Supplementary Table 12B Tabulation of read alignment statistics for Novel variants.

## Abbreviations

COVID-19: coronavirus disease 2019
SARS-CoV-2: severe acute respiratory syndrome coronavirus 2
GISAID: Global Initiative on Sharing All Influenza Data.
