# Supplementary figures and images for: Initial Insights Into the Genetic Epidemiology of SARS-CoV-2 Isolates From Kerala Suggest Local Spread From Limited Introductions
Source: Front Genet. 2021 Mar 17;12:630542. doi: 10.3389/fgene.2021.630542 (PMC8010186; doi:10.3389/fgene.2021.630542)

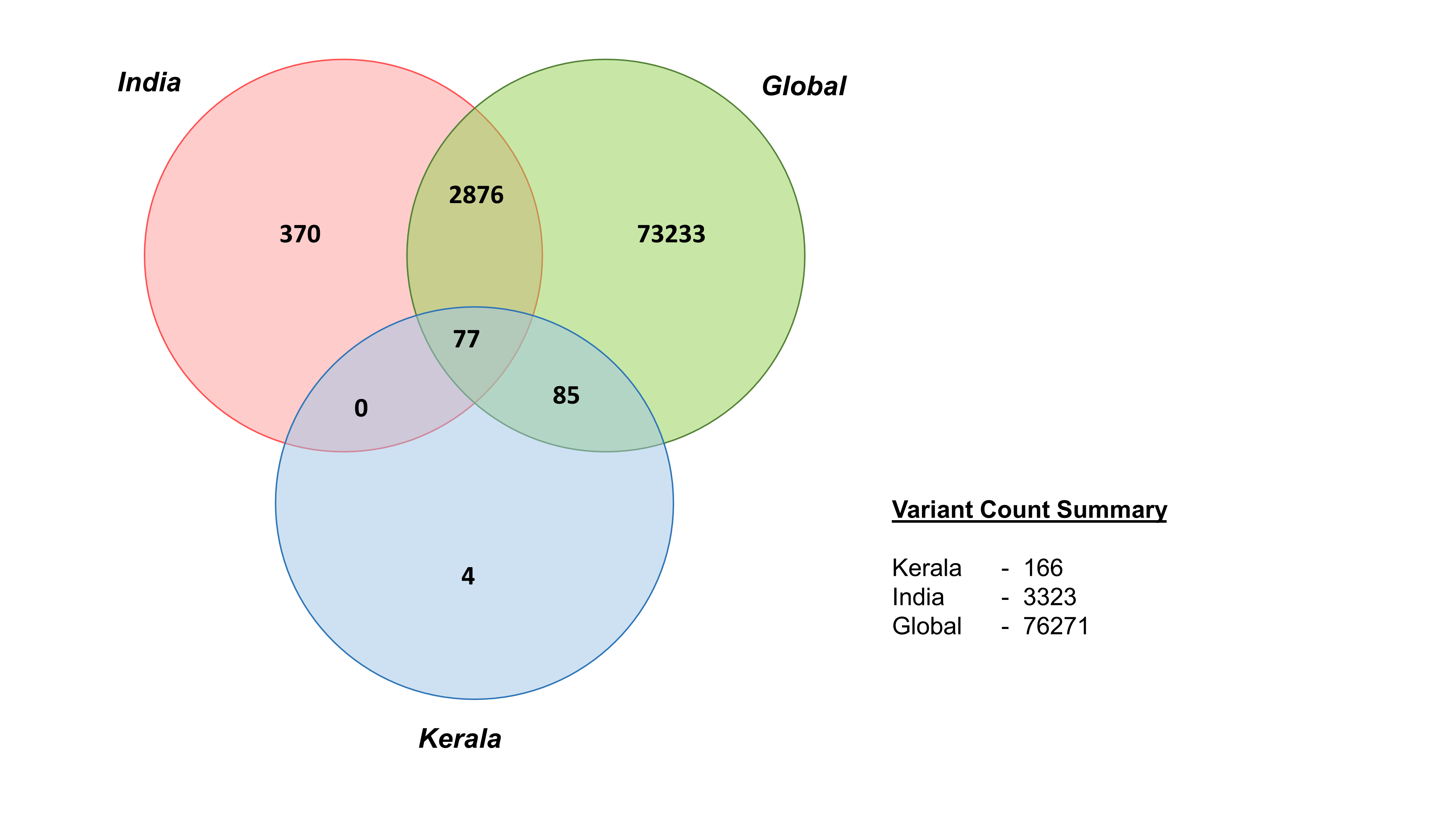

Supplement: Supplementary Figure 1 — Distribution of genetic variants across datasets. [file Image_1.tif]
