# Supplementary material for: Initial Insights Into the Genetic Epidemiology of SARS-CoV-2 Isolates From Kerala Suggest Local Spread From Limited Introductions
Source: Front Genet. 2021 Mar 17;12:630542. doi: 10.3389/fgene.2021.630542 (PMC8010186; doi:10.3389/fgene.2021.630542)
Supplement: Supplementary Table 6 — Compilation of genetic variants identified for the first time in Indian genomes. [file Data_Sheet_3.PDF]

CS Ids	Total Reads	Trimmed Reads q30-30bp	Hisat2 Human %	Hisat2 Covidall %	Hisat2 Unmapped Covid %	BasePairescovered	X coverage	Genome coverage	# bases with 0 coverage	# bases with >0 coverage	# bases with <10x coverage	# bases with <100x coverage	BCFtools Variant Count	VARSCAN Variant Count	N's
CS1896	6011005	5696623	20.20%	76.75%	96.18%	155440637	5198.161957	99.84616928	46	29857	148	2133	10	10	799
CS1897	3951208	3755716	1.22%	97.60%	98.80%	130290961	4357.120055	99.84282513	47	29856	58	102	11	10	15
CS1898	6749650	6409736	4.10%	94.47%	98.51%	215286007	7199.478547	99.97324683	8	29895	57	250	9	8	54
CS1899	6045747	5756524	8.00%	90.50%	98.37%	185230488	6194.378089	99.82944855	51	29852	58	1359	14	13	272
CS1900	4188624	3959355	34.80%	58.19%	89.25%	81925310	2739.702037	99.76256563	71	29832	393	2630	14	13	1419
CS1901	8009923	7308698	5.78%	92.52%	98.19%	240141940	8030.697254	99.91974049	24	29879	71	300	14	14	66
CS1902	8737569	8301051	5.61%	92.98%	98.51%	274362091	9175.06909	99.83613684	49	29854	58	373	14	13	18
CS1903	12831321	12167248	0.72%	98.44%	99.16%	425773231	14238.47878	99.88295489	35	29868	52	100	17	16	17
CS1068	5353173	5053420	32.68%	63.29%	94.02%	113711713	3802.685784	99.64217637	107	29796	929	3364	12	9	1023
CS1069	5084361	4760062	65.69%	9.43%	27.47%	15952745	533.4830953	85.72384042	4269	25634	7675	17118	11	9	7210
CS1804	8836918	7607173	4.16%	94.59%	98.70%	255217334	8534.840451	99.88295489	35	29868	51	75	15	16	122
CS1070	5676866	5292439	9.64%	88.77%	98.24%	166968026	5583.654683	99.91974049	24	29879	393	1968	8	8	683
CS1071	6548817	5745212	1.76%	8.50%	8.65%	17327554	579.4587165	88.07811925	3565	26338	6539	13378	7	6	6028
CS1072	4148686	3907847	1.99%	95.94%	97.88%	133299540	4457.731331	99.36795639	189	29714	938	4707	8	8	1301
CS1073	4645896	4369665	1.88%	97.30%	99.17%	151167324	5055.256128	99.81607197	55	29848	92	1671	11	10	367
CS1074	4855750	4584089	2.91%	96.12%	98.99%	156665045	5239.107949	99.86623416	40	29863	308	2478	10	9	905
CS1075	5457552	5142926	61.40%	31.30%	81.11%	57243911	1914.320001	91.14135705	2649	27254	4429	8295	13	9	4484
CS1076	6331330	5990434	2.10%	96.79%	98.87%	206117902	6892.883724	99.86289001	41	29862	52	80	17	16	15
CS1077	5627062	4874173	0.69%	98.48%	99.17%	170276753	5694.303347	99.84616928	46	29857	53	93	16	16	202
CS1078	5931493	5597265	10.04%	87.76%	97.55%	174629832	5839.876668	99.90970806	27	29876	83	819	15	14	340
CS1079	7635416	7187029	2.12%	32.96%	33.68%	84226986	2816.673444	99.82276026	53	29850	161	1457	11	10	362
CS1805	8008259	7063135	0.13%	99.11%	99.23%	248479526	8309.518309	99.9565261	13	29890	51	164	16	14	39
CS1080	4946581	4665656	8.21%	90.68%	98.79%	150440224	5030.940842	99.93311708	20	29883	343	2216	9	9	807
CS1081	5004390	4720294	12.33%	86.11%	98.22%	144536669	4833.517339	99.74918904	75	29828	1472	5217	9	9	1763
CS1082	5716922	5345590	6.38%	92.00%	98.27%	174788616	5845.186637	99.8261044	52	29851	88	1110	10	10	276
CS1083	5144846	4640554	3.76%	95.23%	98.95%	156947713	5248.56078	99.7893188	63	29840	763	4066	9	9	877
CS1084	5853921	5531509	9.99%	87.20%	96.88%	171518037	5735.813698	99.81941611	54	29849	201	2918	10	9	759
CS1085	8326835	7794762	0.04%	25.15%	25.16%	69700927	2330.900813	99.75922148	72	29831	927	3296	12	11	934
CS1086	5352428	4932432	8.52%	90.26%	98.66%	158161320	5289.145571	99.87961074	36	29867	64	1198	14	13	222
CS1087	5533961	5222671	27.32%	70.04%	96.37%	130056922	4349.293449	99.52178711	143	29760	914	2743	11	11	1228
CS1088	6495097	6098081	7.53%	18.03%	19.50%	39085379	1307.072167	99.12383373	262	29641	4024	7596	12	9	4069
CS1089	6069624	5730152	5.90%	91.93%	97.69%	187327751	6264.513627	99.83613684	49	29854	184	2398	11	10	1097
CS1806	11267752	10211194	2.84%	96.27%	99.09%	349056913	11672.97305	99.84951343	45	29858	54	93	15	14	26
CS1090	6455867	6071875	1.20%	97.71%	98.90%	210883755	7052.26081	99.85620172	43	29860	53	81	11	11	152
CS1091	5716735	5381654	9.43%	88.46%	97.67%	169215131	5658.801157	99.95318195	14	29889	59	514	12	12	226
CS1092	5996865	5562880	2.32%	96.77%	99.06%	191303763	6397.477277	99.85954586	42	29861	72	213	13	13	282
CS1093	6437354	6046923	0.50%	98.74%	99.24%	212236427	7097.496138	99.88295489	35	29868	52	195	11	11	233
CS1094	6023960	5691668	0.53%	98.78%	99.31%	199869839	6683.939371	99.85285757	44	29859	57	90	16	15	110
CS1095	6228033	5877373	3.63%	95.36%	98.95%	199264501	6663.695984	99.88964318	33	29870	76	1500	10	9	298
CS1096	5517511	5200528	1.80%	97.43%	99.21%	180128186	6023.749657	99.85285757	44	29859	56	1062	10	9	274
CS1097	4627636	4372053	14.48%	82.68%	96.68%	128514775	4297.7218	99.97324683	8	29895	127	2026	10	10	390
CS1098	5736631	5403213	31.36%	66.11%	96.32%	127000297	4247.075444	99.61207906	116	29787	1126	4086	11	11	1697
CS1099	5940682	5602790	23.12%	74.29%	96.63%	147982878	4948.763602	99.84282513	47	29856	567	3202	10	10	879
CS1807	6423970	5970262	1.10%	98.06%	99.15%	208018897	6956.455774	99.85285757	44	29859	51	105	15	15	267
CS1100	6021015	5595798	12.49%	85.63%	97.86%	170293566	5694.865599	99.71240344	86	29817	189	4125	11	10	1144
CS1101	5032264	4750842	6.50%	92.64%	99.07%	156481436	5232.967796	99.7458449	76	29827	937	4389	13	11	1034
CS1102	5361918	5067310	2.92%	95.85%	98.73%	172693787	5775.132495	99.92308464	23	29880	94	1463	12	12	327
CS1103	5945123	5619237	0.59%	98.71%	99.30%	197216753	6595.216299	99.84616928	46	29857	75	350	10	10	260
CS1104	6361163	5992041	1.45%	97.59%	99.02%	207870336	6951.487677	99.80269538	59	29844	143	861	12	12	378
CS1105	5096896	4804246	1.78%	97.10%	98.85%	165852528	5546.350801	99.82944855	51	29852	65	1460	11	11	348
CS1106	5781113	5448832	0.87%	98.33%	99.19%	190494249	6370.405946	99.9130522	26	29877	62	1504	8	8	316
CS1107	5396943	5080641	1.58%	96.32%	97.87%	174016666	5819.371501	99.69233856	92	29811	493	4359	17	16	1496
CS1108	5119471	4813989	14.20%	83.81%	97.68%	143408259	4795.781661	99.92977293	21	29882	361	3224	14	13	727

CS1109	5392725	5091620	13.66%	81.39%	94.27%	147333203	4927.037521	99.83613684	49	29854	113	1863	10	10	363
CS1808	11226700	10614989	0.95%	98.29%	99.23%	370901392	12403.48433	99.81941611	54	29849	61	244	12	11	11
CS1110	5443316	5104358	5.40%	93.31%	98.64%	169285860	5661.166438	99.83948099	48	29855	64	820	9	9	226
CS1111	5449833	5135172	16.20%	78.41%	93.57%	143143368	4786.923319	99.89967562	30	29873	88	1587	11	11	324
CS1112	5131904	4816260	2.46%	96.72%	99.15%	165569961	5536.901348	99.73915661	78	29825	90	343	10	10	46
CS1113	7036316	6621483	0.96%	98.35%	99.30%	231471965	7740.760626	99.83948099	48	29855	61	258	9	9	16
CS1114	4803614	4520552	14.53%	83.96%	98.23%	134950864	4512.954018	99.65220881	104	29799	749	4769	10	10	1432
CS1115	5196983	4898428	39.15%	56.17%	92.31%	97820153	3271.248804	98.92987326	320	29583	2331	6409	10	9	2652
CS1116	5771368	5456060	24.28%	73.53%	97.10%	142636790	4769.98261	99.07367154	277	29626	1066	3366	10	8	1950
CS1117	5397037	4348379	13.02%	85.49%	98.28%	131736107	4405.447848	99.5719493	128	29775	885	1373	15	13	1276
CS1118	5973371	5619215	34.66%	62.30%	95.33%	124454663	4161.945725	99.32782664	201	29702	1300	3145	10	9	2023
CS1119	5039736	4750391	16.93%	81.32%	97.89%	137352445	4593.266395	99.60873491	117	29786	1488	4874	10	9	1457
CS1809	7327292	6956925	17.21%	79.31%	95.80%	196165506	6560.061064	99.89633147	31	29872	139	565	12	11	361
CS1120	10145	6364	0.08%	4.05%	4.06%	7542	0.2522154968	0.5216867873	29747	156	29843	29872	#N/A	#N/A	16042
CS1121	5049521	4315914	2.66%	81.48%	83.70%	124732984	4171.253185	99.8327927	50	29853	74	1171	15	15	282
CS1122	6270434	5885722	5.07%	93.78%	98.78%	196182168	6560.618266	99.85954586	42	29861	50	367	16	15	269
CS1123	5133354	4843774	0.97%	98.37%	99.33%	169405164	5665.156138	99.81607197	55	29848	88	987	15	14	295
CS1124	6356455	5854244	2.14%	96.93%	99.04%	201569858	6740.790489	99.87961074	36	29867	63	226	13	13	292
CS1125	5542178	5161203	3.70%	95.22%	98.88%	174672459	5841.302177	99.83948099	48	29855	99	1461	14	14	298
CS1126	5035121	4655388	3.65%	94.52%	98.10%	156346481	5228.454704	99.91639635	25	29878	86	1038	13	13	336
CS1127	5709455	4769793	2.69%	95.92%	98.58%	162227019	5425.108484	99.84616928	46	29857	73	879	13	13	233
CS1128	6219490	5862076	21.45%	76.52%	97.42%	159479982	5333.243554	99.8261044	52	29851	478	2348	9	9	815
CS1129	4102886	3879629	1.97%	97.26%	99.21%	134115116	4485.005384	99.85285757	44	29859	60	103	10	10	55
CS1810	10392691	9838901	1.65%	97.52%	99.16%	341062908	11405.64184	99.8261044	52	29851	65	258	11	11	17
CS1130	5420458	5120076	5.84%	93.11%	98.88%	169490605	5668.01341	99.91639635	25	29878	90	1511	12	12	394
CS1131	6012270	5692963	2.63%	96.51%	99.12%	195356578	6533.00933	99.84616928	46	29857	57	693	10	9	278
CS1132	4857107	4574339	23.31%	74.38%	96.99%	120968354	4045.358459	99.87961074	36	29867	231	611	9	10	1010
CS1133	5128420	4718889	17.75%	80.84%	98.28%	135554087	4533.126676	99.69902685	90	29813	521	5670	9	10	1594
CS1134	7377548	6971926	5.18%	93.95%	99.09%	232902756	7788.608367	99.85620172	43	29860	85	857	10	10	307
CS1135	6159498	5791815	16.01%	38.43%	45.75%	79148470	2646.840451	95.17439722	1443	28460	4522	7427	10	11	4180
CS1136	5949510	5595842	10.16%	87.92%	97.87%	174881623	5848.296927	99.92642879	22	29881	62	1077	12	13	341
CS1137	6140108	5798311	3.54%	95.64%	99.15%	197153667	6593.106611	99.85285757	44	29859	75	189	15	14	280
CS1138	5636748	5322714	9.19%	88.45%	97.40%	167377449	5597.346387	99.93980537	18	29885	75	523	15	15	319
CS1139	6614597	6164809	47.60%	33.00%	62.98%	72326653	2418.708926	97.1474434	853	29050	3333	6929	15	13	3173
CS1811	8290150	7852724	3.80%	95.09%	98.85%	265462483	8877.453199	99.84282513	47	29856	54	102	14	14	139
CS1140	6151367	5809823	0.36%	98.49%	98.85%	203412925	6802.425342	99.89298733	32	29871	51	322	12	12	279
CS1141	6046396	5705075	11.06%	87.52%	98.41%	177519204	5936.501488	99.7458449	76	29827	331	1316	11	10	504
CS1142	6405889	6018588	8.14%	90.71%	98.76%	194080646	6490.3403	99.83948099	48	29855	77	1350	10	10	297
CS1143	4084746	3845510	5.62%	93.30%	98.85%	127548315	4265.401966	99.81607197	55	29848	76	519	10	10	278
CS1144	8004094	7567378	3.32%	95.69%	98.97%	257382610	8607.250443	99.8327927	50	29853	58	365	10	10	187
CS1145	6768381	6400431	3.30%	95.78%	99.05%	217911639	7287.283517	99.94649366	16	29887	56	113	17	16	90
CS1146	4999181	4510776	2.23%	93.66%	95.79%	149992273	5015.960706	99.96990269	9	29894	59	1309	11	11	272
CS1147	5062479	4692223	8.65%	61.07%	66.86%	101845328	3405.856536	99.87292245	38	29865	1075	3190	11	11	954
CS1148	5372254	5027203	4.53%	94.61%	99.10%	169061261	5653.65552	99.8695783	39	29864	86	968	10	10	312
CS1149	4968156	4698955	9.66%	89.13%	98.66%	148900185	4979.439688	99.8261044	52	29851	336	1971	11	11	712
CS1812	7551226	6898516	1.90%	97.26%	99.14%	238293270	7968.875029	99.85620172	43	29860	57	429	15	14	211
CS1150	5890375	5495519	14.71%	83.85%	98.32%	163773974	5476.840919	99.7893188	63	29840	737	4036	12	10	721
CS1151	7104529	6667738	1.28%	98.03%	99.30%	232305403	7768.63201	99.84282513	47	29856	55	107	11	11	20
CS1152	5522232	5199706	6.04%	92.54%	98.49%	171048639	5720.116343	99.88629903	34	29869	91	939	11	11	304
CS1153	6480588	6021794	1.54%	97.77%	99.30%	209213620	6996.409056	99.82944855	51	29852	89	1084	11	11	295
CS1154	5849220	5516893	7.92%	90.97%	98.79%	178418451	5966.573621	99.75922148	72	29831	138	1317	12	12	320
CS1155	5884203	5550712	1.06%	98.30%	99.35%	193983744	6487.099756	99.81272782	56	29847	88	705	12	12	265
CS1156	6099940	5317785	2.98%	96.02%	98.98%	181183179	6059.030164	99.95318195	14	29889	75	367	11	11	73
CS1157	5245255	4943017	18.57%	79.59%	97.74%	139871507	4677.507508	99.80269538	59	29844	457	2122	11	10	767
CS1158	5648901	4852302	21.09%	75.68%	95.91%	130284376	4356.899843	99.81272782	56	29847	1191	4543	12	11	1173

CS1159	5287524	4990529	0.81%	98.29%	99.09%	174370230	5831.195198	99.8327927	50	29853	68	1455	11	11	296
CS1813	8206194	7756400	3.68%	95.09%	98.73%	262225751	8769.212153	99.72578002	82	29821	145	1052	13	13	1464
CS1795	14245366	13509456	0.58%	98.34%	98.91%	472165213	15789.89443	99.85285757	44	29859	50	57	10	10	11
CS1160	6548309	6172648	1.47%	97.54%	98.99%	214005381	7156.652543	99.96990269	9	29894	64	153	12	11	253
CS1814	9563688	9028069	1.13%	98.19%	99.31%	315070739	10536.42574	99.8327927	50	29853	89	193	14	13	160
CS1815	10019608	9523894	1.30%	97.97%	99.26%	331691180	11092.23757	99.8261044	52	29851	83	99	14	14	75
CS1816	9664860	9055485	2.31%	96.83%	99.12%	311597712	10420.28265	99.8695783	39	29864	57	101	14	13	44
CS1817	13264607	12550591	0.35%	98.92%	99.26%	441380095	14760.39511	99.94649366	16	29887	43	56	14	14	29
CS1818	9879870	8928739	0.38%	98.94%	99.32%	313668287	10489.5257	99.81272782	56	29847	88	107	12	12	37
CS1819	9909813	9388142	0.88%	98.30%	99.16%	328026414	10969.68244	99.87961074	36	29867	53	83	14	13	27
CS1820	5479996	5096243	19.00%	77.53%	95.72%	140371770	4694.237033	99.5719493	128	29775	226	1259	8	8	1808
CS1821	13570547	12878688	1.24%	97.90%	99.13%	448145526	14986.64101	99.86289001	41	29862	49	70	14	14	24
CS1822	13849926	11640395	2.05%	96.88%	98.91%	399920049	13373.91061	99.83948099	48	29855	85	560	8	8	237
CS1823	16688459	15856965	0.63%	98.63%	99.25%	555999835	18593.44664	99.81607197	55	29848	83	112	11	11	51
CS1796	1482384	1383619	87.94%	8.31%	68.90%	4085435	136.6229141	6.464234358	27970	1933	28167	28293	1	1	28200
CS1824	16889111	16043578	1.72%	97.29%	98.99%	554904623	18556.82116	99.83948099	48	29855	53	96	11	10	10
CS1825	21250504	20094373	19.94%	72.08%	90.03%	514940775	17220.3717	99.96655854	10	29893	49	531	11	10	227
CS1826	20228344	17700371	4.87%	93.94%	98.75%	589966901	19729.35495	99.95987025	12	29891	82	258	11	11	79
CS1827	16424777	15630659	19.73%	76.03%	94.72%	422534241	14130.16222	99.92308464	23	29880	217	641	11	11	209
CS1828	9365061	8864461	1.96%	96.88%	98.82%	305234065	10207.473	99.79935124	60	29843	208	255	10	10	156
CS1829	9742635	8563813	0.60%	98.53%	99.13%	299470293	10014.72404	99.87292245	38	29865	53	67	14	13	19
CS1830	13027402	12348495	1.74%	97.29%	99.01%	427035847	14280.7025	99.86289001	41	29862	49	65	13	12	10
CS1831	11622127	11006082	3.40%	94.63%	97.97%	370285987	12382.90429	99.96990269	9	29894	91	258	12	12	75
CS1832	11566356	10885741	2.46%	96.65%	99.09%	373887270	12503.33645	99.84282513	47	29856	59	214	12	11	104
CS1833	11070971	9829766	16.37%	76.16%	91.07%	265730674	8886.421897	99.92308464	23	29880	217	704	12	12	285
CS1797	7209742	6674603	1.93%	97.25%	99.16%	230586562	7711.151456	99.85285757	44	29859	56	108	15	15	101
CS1836	10711499	10162836	2.37%	96.57%	98.91%	348887734	11667.31545	99.84616928	46	29857	53	78	13	13	16
CS1837	12965575	12285708	7.46%	88.87%	96.03%	388100114	12978.63472	99.9565261	13	29890	30	258	11	12	30
CS1838	14720595	13817560	13.84%	84.06%	97.56%	412767884	13803.56098	99.97324683	8	29895	54	217	11	10	227
CS1839	14124594	12516982	10.53%	86.89%	97.11%	385964710	12907.22369	99.97993512	6	29897	71	282	11	11	73
CS1840	16023748	15167013	3.45%	94.67%	98.05%	510448741	17070.15152	99.96655854	10	29893	26	244	12	12	55
CS1841	18245950	17280821	4.31%	94.50%	98.76%	580488786	19412.39294	99.87961074	36	29867	50	85	9	9	68
CS1842	22238804	21142118	5.16%	93.60%	98.69%	703486750	23525.62452	99.98327927	5	29898	33	143	10	8	68
CS1843	25088909	23842703	3.57%	95.32%	98.85%	807762809	27012.76825	99.88964318	33	29870	45	56	13	13	14
CS1844	6365048	6055513	1.96%	97.14%	99.08%	209167866	6994.878975	99.8261044	52	29851	158	985	14	12	595
CS1845	7386514	6453383	21.52%	73.36%	93.47%	167972616	5617.249641	99.94649366	16	29887	74	1027	14	14	355
CS1798	6689743	6340321	5.61%	93.37%	98.92%	210450170	7037.761094	99.8261044	52	29851	61	664	12	12	210
CS1846	9802962	9310696	8.71%	89.57%	98.12%	296508334	9915.671806	99.97659098	7	29896	58	416	15	14	242
CS1847	13310793	12631620	11.45%	85.32%	96.35%	383110626	12811.77895	99.94314952	17	29886	76	391	12	11	71
CS1848	13782250	13039460	18.23%	72.86%	89.11%	337707649	11293.43708	99.95987025	12	29891	45	280	9	9	109
CS1849	4705257	4462810	0.00%	97.88%	97.88%	155335201	5194.636023	37.33070261	18740	11163	19554	19606	4	4	25428
CS1850	17808366	16748123	12.08%	86.20%	98.04%	513276753	17164.72438	99.63883222	108	29795	444	1716	9	9	679
CS1851	17069993	15512013	12.66%	85.59%	97.99%	471415879	15764.8356	99.95987025	12	29891	62	159	10	10	240
CS1852	4741354	4484460	62.29%	19.57%	51.90%	31210163	1043.71344	97.98347992	603	29300	3630	9455	11	10	3652
CS1853	7053975	6674559	2.54%	96.30%	98.81%	228445499	7639.551182	99.92977293	21	29882	59	109	15	13	55
CS1854	6472320	5993896	1.28%	97.57%	98.83%	207738046	6947.063706	99.84616928	46	29857	76	292	16	15	198
CS1855	7352891	6973484	2.21%	89.99%	92.03%	223085915	7460.318864	99.85954586	42	29861	61	474	14	13	225
CS1799	7997214	7536700	3.40%	95.68%	99.06%	256310386	8571.393706	99.84951343	45	29858	55	100	12	12	47
CS1856	10209896	9654205	4.11%	93.55%	97.56%	320966404	10733.58539	99.98662342	4	29899	52	93	11	11	181
CS1857	19995846	18898935	0.20%	11.79%	11.81%	79200672	2648.586162	98.78273083	364	29539	2143	5576	11	10	2406
CS1858	13661481	12910290	9.35%	88.85%	98.01%	407807807	13637.68876	99.81272782	56	29847	202	1836	10	10	832
CS1859	13824886	13082875	4.24%	93.47%	97.61%	434635235	14534.83714	99.85285757	44	29859	61	224	10	10	65
CS1860	4754606	4443090	14.15%	83.83%	97.65%	132358321	4426.255593	99.67896198	96	29807	347	2102	13	13	549
CS1861	5801320	5482161	9.91%	88.19%	97.88%	171828670	5746.201719	99.93311708	20	29883	202	1040	13	13	379
CS1862	8846456	8351640	0.42%	11.44%	11.49%	33968313	1135.950005	94.34170485	1692	28211	4575	7732	10	10	4252

CS1863	6978541	6623326	17.23%	80.51%	97.27%	189616735	6341.060596	99.81941611	54	29849	129	1587	11	11	494
CS1864	9743342	9241040	2.21%	96.18%	98.36%	315950650	10565.85125	99.87961074	36	29867	65	137	8	8	38
CS1865	7890781	7507101	3.99%	84.65%	88.17%	225959821	7556.426479	99.81941611	54	29849	59	878	14	14	277
CS1800	9138561	8613764	0.83%	98.30%	99.13%	300972721	10064.96743	99.84616928	46	29857	59	103	13	13	48
CS1866	10739141	10174272	28.81%	66.49%	93.40%	240520038	8043.341404	99.77594221	67	29836	249	1112	14	12	504
CS1867	12067090	11440050	2.85%	96.01%	98.82%	390429609	13056.53643	99.97324683	8	29895	56	101	12	12	60
CS1868	7811122	7239328	1.67%	97.00%	98.65%	249448349	8341.917166	99.87292245	38	29865	55	100	14	13	22
CS1869	9222564	8752168	0.13%	99.13%	99.26%	308420713	10314.03916	99.95318195	14	29889	53	83	14	13	43
CS1870	8114031	7714832	1.65%	97.39%	99.03%	267111956	8932.613985	99.84282513	47	29856	58	95	16	16	162
CS1871	8422981	7999844	14.02%	82.84%	96.35%	235632994	7879.911514	99.97659098	7	29896	37	499	16	14	277
CS1872	12723229	12055008	5.51%	92.87%	98.29%	397993588	13309.48694	99.97993512	6	29897	23	80	15	15	249
CS1873	8915258	8450913	4.72%	91.87%	96.42%	275938635	9227.791024	99.98327927	5	29898	56	163	15	15	77
CS1874	9904191	9379599	24.54%	68.35%	90.57%	227922632	7622.065746	99.96321439	11	29892	58	1205	16	15	313
CS1875	8236175	7810987	15.99%	81.53%	97.04%	226392674	7570.901716	99.81607197	55	29848	65	669	11	10	274
CS1801	9030161	8563297	17.64%	75.76%	91.98%	230680680	7714.2989	99.90970806	27	29876	197	921	11	11	305
CS1876	4376255	4141672	20.09%	76.59%	95.84%	112781465	3771.576932	99.90301976	29	29874	424	2164	11	9	890
CS1877	7613296	7235916	0.56%	98.55%	99.11%	253494590	8477.229375	99.8762666	37	29866	54	79	15	14	31
CS1878	9312963	8778144	0.23%	98.89%	99.12%	308487553	10316.27439	99.87292245	38	29865	51	65	15	14	29
CS1879	17070963	16192229	0.57%	98.73%	99.30%	568283166	19004.21918	99.85954586	42	29861	49	58	12	11	9
CS1880	11352159	10738152	3.49%	95.55%	99.00%	364738383	12197.38431	99.85285757	44	29859	58	365	9	9	226
CS1881	12679877	12018730	23.01%	74.83%	97.20%	319803200	10694.68615	99.75253319	74	29829	88	1067	9	9	619
CS1882	12863553	12180097	91.01%	3.13%	34.83%	13565201	453.6401364	18.83757483	24270	5633	25890	26280	5	2	25735
CS1883	14694899	13968401	8.10%	90.42%	98.39%	449006435	15015.43106	99.82944855	51	29852	70	318	10	10	272
CS1884	6374062	6052916	2.08%	96.94%	99.01%	208637897	6977.156038	99.82276026	53	29850	134	1581	11	11	537
CS1885	6867035	5544600	10.10%	87.20%	96.99%	171315991	5729.056984	99.8261044	52	29851	67	1007	11	11	172
CS1802	10169376	9563454	6.21%	90.74%	96.75%	308472704	10315.77781	99.92977293	21	29882	138	366	8	8	115
CS1886	7407713	6998256	1.46%	29.36%	29.79%	73037709	2442.487677	99.41143029	176	29727	1417	5177	11	11	1479
CS1887	7484378	7097486	2.93%	95.83%	98.72%	241794536	8085.962479	99.88295489	35	29868	63	224	11	11	29
CS1888	9688673	9190783	2.63%	96.26%	98.86%	314527406	10518.25589	99.86289001	41	29862	52	90	12	12	191
CS1889	8262427	7126058	3.42%	95.21%	98.58%	240670956	8048.388322	99.84282513	47	29856	52	84	11	11	271
CS1890	9920499	9365888	1.72%	96.72%	98.42%	321934613	10765.96372	99.88295489	35	29868	48	63	11	11	199
CS1891	8739719	8290784	4.60%	82.11%	86.07%	242057737	8094.764305	99.84951343	45	29858	59	360	9	8	339
CS1892	5302251	4922068	2.07%	96.94%	98.99%	169511948	5668.727151	99.83613684	49	29854	56	1228	12	12	241
CS1893	5822983	5456387	8.25%	90.44%	98.58%	175403279	5865.741865	99.95318195	14	29889	59	1215	13	12	365
CS1894	4630806	4307798	6.16%	91.97%	98.01%	140779644	4707.876935	99.81272782	56	29847	86	648	15	14	296
CS1895	6033648	5138672	18.31%	79.19%	96.94%	144359596	4827.59576	97.07387219	875	29028	1694	3176	17	16	2661
CS1803	9918776	9402455	1.15%	97.94%	99.08%	327320719	10946.08297	99.85954586	42	29861	49	61	15	14	12
