# Supplementary material for: Initial Insights Into the Genetic Epidemiology of SARS-CoV-2 Isolates From Kerala Suggest Local Spread From Limited Introductions
Source: Front Genet. 2021 Mar 17;12:630542. doi: 10.3389/fgene.2021.630542 (PMC8010186; doi:10.3389/fgene.2021.630542)
Supplement: Supplementary Table 7 — Unique haplotypes for the dataset of 850 genomes belonging to clade A2a. [file Data_Sheet_4.PDF]

Chr	Pos	Ref	Alt	Occurrence	Average Variation Percentage
1	2334	C	T	1	100
1	2523	C	T	1	100
1	2875	G	T	1	100
1	3604	C	T	1	100
1	3728	G	A	4	100
1	4754	C	T	1	100
1	5812	C	G	1	100
1	5833	C	T	1	100
1	5866	C	T	1	100
1	6402	C	T	1	100
1	8371	G	T	4	100
1	8782	C	T	1	100
1	9707	A	G	1	100
1	11950	C	T	1	100
1	13957	C	T	1	100
1	14120	C	T	1	100
1	16402	G	T	4	100
1	19017	C	T	1	100
1	20302	G	A	2	100
1	21017	C	T	2	100
1	21575	C	T	28	100
1	21621	C	T	9	100
1	21846	C	T	1	100
1	21952	C	T	1	100
1	22201	G	T	2	100
1	22444	C	T	8	100
1	22448	C	A	3	100
1	22468	G	T	1	100
1	23248	C	T	1	100
1	23719	T	C	2	100
1	24130	C	T	1	100
1	24933	G	T	1	100
1	25511	C	T	1	100
1	25546	C	T	1	100
1	26486	T	A	1	100
1	28024	A	G	1	100
1	28028	G	A	1	100
1	28878	G	A	1	100
1	28980	G	T	1	100
1	29614	C	T	1	100
1	29659	C	T	4	100
1	28854	C	T	6	99.9967
1	16726	C	T	47	99.9962
1	18129	C	T	9	99.9956
1	3653	C	T	9	99.9944
1	12017	T	G	4	99.9925
1	12325	C	T	6	99.9917
1	26735	C	T	6	99.9917
1	2857	C	T	4	99.99
1	4784	C	T	4	99.99
1	12885	C	T	4	99.99
1	17470	C	T	4	99.99
1	9891	C	T	5	99.988
1	11417	G	T	4	99.9875
1	27612	C	T	44	99.9866
1	18246	C	T	4	99.985
1	4936	G	A	47	99.9849
1	16912	G	T	4	99.9825
1	17321	C	T	4	99.9825
1	18496	A	G	4	99.9825
1	14408	C	T	178	99.9824
1	832	C	T	1	99.98
1	9943	C	T	4	99.98
1	25169	C	T	1	99.98
1	26367	G	C	2	99.98
1	23188	C	T	26	99.9788
1	5700	C	A	47	99.9785
1	27110	C	T	38	99.9784
1	12312	C	T	13	99.9777
1	18186	G	A	19	99.9758
1	3087	T	C	2	99.975
1	10039	C	T	2	99.975
1	23403	A	G	178	99.9749
1	23896	C	T	47	99.9749
1	17808	G	T	19	99.9747
1	24794	G	A	26	99.9742
1	8683	C	T	20	99.9735
1	16887	C	T	3	99.9733
1	18412	G	T	4	99.9725
1	8917	C	T	121	99.9707
1	18647	C	T	4	99.97
1	28907	G	T	1	99.97
1	1218	C	T	38	99.9697
1	3037	C	T	178	99.9693
1	1094	T	C	9	99.9678
1	27384	T	C	38	99.9661
1	4904	G	T	2	99.965
1	21071	A	G	2	99.965
1	25563	G	T	6	99.965
1	529	G	T	26	99.9604
1	500	G	A	1	99.96
1	17462	G	A	1	99.96

Chr	Pos	Ref	Alt	Occurrence	Average Variation Percentage
1	17479	G	A	1	99.96
1	20940	G	T	1	99.96
1	25281	G	A	1	99.96
1	11653	C	T	7	99.9571
1	25621	G	T	3	99.9567
1	28948	C	T	3	99.9567
1	241	C	T	177	99.9559
1	9389	G	A	69	99.9555
1	14857	G	T	2	99.955
1	17002	C	T	4	99.955
1	2453	C	T	12	99.9517
1	21077	C	T	2	99.95
1	18877	C	T	6	99.9483
1	5724	C	T	2	99.945
1	21486	T	C	4	99.945
1	1426	C	T	4	99.9425
1	872	G	A	1	99.94
1	25812	T	C	14	99.9379
1	186	C	T	8	99.935
1	20578	G	T	2	99.935
1	11461	C	T	2	99.93
1	13414	T	C	1	99.93
1	16323	C	T	2	99.93
1	27294	C	T	1	99.93
1	313	C	T	47	99.9289
1	4144	G	A	19	99.9216
1	20569	G	T	1	99.92
1	20413	T	C	21	99.9186
1	6355	A	G	1	99.91
1	9448	C	T	1	99.91
1	20703	C	T	1	99.91
1	21008	C	T	1	99.91
1	26933	A	G	1	99.87
1	26113	G	T	1	99.86
1	683	C	T	1	99.85
1	11195	C	T	47	99.8455
1	28899	G	T	2	99.84
1	5822	C	T	5	99.782
1	6294	T	C	6	99.7683
1	27092	C	T	1	99.74
1	16188	G	T	1	99.71
1	4084	C	T	1	99.69
1	27643	C	A	5	99.642
1	936	C	T	2	99.565
1	11619	T	C	2	99.515
1	6070	C	T	1	99.48
1	22032	T	C	2	99.435
1	3871	G	T	45	98.6353
1	25703	C	T	26	98.5604
1	18008	A	G	1	98.54
1	16750	C	T	8	98.4438
1	4201	G	A	5	97.268
1	13085	G	A	1	97.06
1	5907	C	T	1	96.4
1	3787	C	T	1	95.92
1	14874	G	T	2	95.845
1	21933	C	T	1	95.84
1	27493	C	T	1	95.67
1	21974	G	T	2	95.335
1	27213	C	T	4	95.29
1	28085	G	T	1	95.11
1	29675	C	T	4	94.88
1	11083	G	T	7	93.5314
1	10448	C	T	2	91.355
1	16557	T	C	1	88.17
1	19224	T	C	1	87.73
1	5413	C	T	4	84.415
1	11866	C	T	1	74.58
1	22675	C	T	1	63.18
1	19153	A	G	1	62.14
1	21855	C	T	1	60.22
1	1580	G	T	1	58.05
1	28086	G	T	1	50.57
1	15546	C	A	4	50.04
**Variants with insufficient variation percentage**					
1	14178	C	T	1	46.77
1	12473	C	T	1	45.72
1	1519	C	T	1	44.96
1	28895	G	T	1	44.51
1	337	C	T	46	44.4535
1	24374	C	T	1	44.05
1	27672	T	C	1	42.19
1	10465	G	A	1	42.02
1	5051	C	T	1	40.32
1	5974	C	T	1	39.5
1	13517	C	T	1	39.01
1	4148	G	T	1	35.32
1	1437	C	T	1	31.9
1	884	C	T	1	31.18
1	27625	C	T	1	30.26
1	21637	C	T	1	28.16
1	25579	A	T	1	27.69

Chr	Pos	Ref	Alt	Occurrence	Average Variation Percentage
1	29831	C	T	1	27.59
1	26428	G	T	1	26.97
1	14982	T	C	1	24.37
1	3994	G	A	1	24.34
1	2939	C	T	1	23.84
1	5183	C	T	1	22.78
1	346	C	T	1	22.6
1	5290	T	C	1	21.93
1	631	T	C	1	20.61
1	15194	C	T	1	20.5
1	18099	T	A	1	20.29
1	9779	T	C	1	20.26
