# Supplementary material for: Initial Insights Into the Genetic Epidemiology of SARS-CoV-2 Isolates From Kerala Suggest Local Spread From Limited Introductions
Source: Front Genet. 2021 Mar 17;12:630542. doi: 10.3389/fgene.2021.630542 (PMC8010186; doi:10.3389/fgene.2021.630542)
Supplement: Supplementary Table 8 — Mutations unique to the three haplogroups and their frequency in Kerala. [file Data_Sheet_5.PDF]

Chr	Start	End	Ref	Alt	Func.refGene	Gene.refGene	GeneDetail.refG	ExonicFunc.ref(	AAChange.refG	GERP++_RS	PhyoP44wayCo	PhastCons44wa	SIFT_score	SIFT_pred	UNIPROT_Disul	UNIPROT_doma	UNIPROT_glyph	UNIPROT_Trans	b_CellEpitopes	cd4_Epitopes	cd4_Epitope_Sc	cd8_Epitopes	cd8_Epitope_Sc	MPDI_Potential	MPDI_Potential	ARTIC_Primers	RT_PCR-Primer	RT_PCR-Primer	Sequencing_Err	Homoplasic_po	Hypermutable_	Otherinfo
1	186	186	C	T	upstream	ORF1a	dist=80	.	.	1.6	3.21217	1	.	.	.	.	.	.	.	.	.	.	.	.	.	.	.	.	.	.	.	
1	529	529	G	T	exonic	ORF1a	.	synonymous SN	ORF1a:cds-YP_	-2.7	0.677165	0.92126	1	T	.	.	.	.	.	.	.	orf1ab_APHGHV	0.03	.	.	.	.	.	.	.	.	
1	872	872	G	A	exonic	ORF1a	.	nonsynonymous	ORF1a:cds-YP_	0.741	2.76482	1	0.35	T	.	.	.	.	.	.	.	.	.	.	.	.	.	.	.	.	.	
1	936	936	C	T	exonic	ORF1a	.	nonsynonymous	ORF1a:cds-YP_	1.65	3.28673	1	0.05	D	.	.	.	.	.	.	.	orf1ab_FIDTKRC	0.05	.	.	.	.	.	.	.	.	
1	1580	1580	G	T	exonic	ORF1a	.	nonsynonymous	ORF1a:cds-YP_	-1.83	0.154331	0.992126	0.04	D	.	.	.	.	.	.	.	.	.	.	.	nCoV-2019_6_L	.	.	.	.	.	
1	2523	2523	C	T	exonic	ORF1a	.	nonsynonymous	ORF1a:cds-YP_	1.65	3.34816	0.645669	0.01	D	.	.	.	.	.	.	.	.	.	.	.	nCoV-2019_9_L	.	.	.	.	.	
1	2875	2875	G	T	exonic	ORF1a	.	nonsynonymous	ORF1a:cds-YP_	-3.3	-0.43452	0.96063	0.59	T	.	.	.	.	.	.	.	.	.	.	.	.	.	.	.	.	.	
1	3087	3087	T	C	exonic	ORF1a	.	nonsynonymous	ORF1a:cds-YP_	-3.03	-0.244354	0	0.41	T	.	.	.	.	.	.	.	orf1ab_GDCEEE	-1	.	.	.	.	.	.	.	.	
1	3653	3653	C	T	exonic	ORF1a	.	nonsynonymous	ORF1a:cds-YP_	0.72	1.90197	0.992126	0.01	D	.	Macro	.	.	.	.	.	.	.	.	.	.	.	.	.	.	.	
1	3728	3728	G	A	exonic	ORF1a	.	nonsynonymous	ORF1a:cds-YP_	1.65	4.256	1	0.33	T	.	Macro	.	.	.	orf1ab polyprotei	15	.	.	.	.	.	.	.	.	.	.	
1	3787	3787	C	T	exonic	ORF1a	.	synonymous SN	ORF1a:cds-YP_	-3.3	-1.49061	0.015748	1	T	.	Macro	.	.	.	.	.	.	.	.	.	nCoV-2019_13_	.	.	.	.	.	
1	3871	3871	G	T	exonic	ORF1a	.	nonsynonymous	ORF1a:cds-YP_	-3.3	3.28669	0.992126	0.24	T	.	.	.	.	.	.	.	orf1ab_SEKQVE	-1	.	.	.	.	.	.	.	.	
1	4144	4144	G	A	exonic	ORF1a	.	synonymous SN	ORF1a:cds-YP_	-2.53	-0.0520236	0.937008	1	T	.	.	.	.	.	.	.	.	.	.	.	nCoV-2019_13_	.	.	.	.	.	
1	4201	4201	G	A	exonic	ORF1a	.	nonsynonymous	ORF1a:cds-YP_	1.65	4.256	1	0.18	T	.	.	.	.	.	.	.	.	.	.	.	.	.	.	.	.	.	
1	4754	4754	C	T	exonic	ORF1a	.	nonsynonymous	ORF1a:cds-YP_	-3.3	-0.539724	0.716535	0.55	T	.	.	.	.	.	.	.	.	.	.	.	.	.	.	.	.	.	
1	4784	4784	C	T	exonic	ORF1a	.	nonsynonymous	ORF1a:cds-YP_	-3.18	-0.214591	0.0472441	0.06	T	.	.	.	.	.	.	.	orf1ab_TISLAGS	-1	.	.	.	.	.	.	.	.	
1	4936	4936	G	A	exonic	ORF1a	.	synonymous SN	ORF1a:cds-YP_	-3.3	0.679528	0.976378	1	T	.	.	.	.	.	orf1ab polyprotei	9.2	orf1ab_NLKTLLS	0.08	.	.	.	.	.	.	.	.	
1	5413	5413	C	T	exonic	ORF1a	.	synonymous SN	ORF1a:cds-YP_	-3.3	-0.341465	0.984252	1	T	.	Peptidase_C16	.	.	.	.	.	.	.	.	.	.	.	.	.	.	.	
1	5724	5724	C	T	exonic	ORF1a	.	nonsynonymous	ORF1a:cds-YP_	0.701	1.5908	0.913386	0.28	T	.	Peptidase_C16	.	.	.	.	.	.	.	.	.	.	.	.	.	.	.	
1	5812	5812	C	G	exonic	ORF1a	.	nonsynonymous	ORF1a:cds-YP_	-3.3	-0.408094	0.984252	0	D	.	Peptidase_C16	.	.	.	.	.	.	.	.	.	.	.	.	.	.	.	
1	5833	5833	C	T	exonic	ORF1a	.	synonymous SN	ORF1a:cds-YP_	-0.546	0.724614	0.992126	0.32	T	.	Peptidase_C16	.	.	.	.	.	orf1ab_SEYKGP	-1	.	.	.	.	.	.	.	.	
1	5866	5866	C	T	exonic	ORF1a	.	synonymous SN	ORF1a:cds-YP_	1.65	3.32318	1	0.48	T	.	Peptidase_C16	.	.	.	.	.	orf1ab_SEYKGP	-1	.	.	.	.	.	.	.	.	
1	5907	5907	C	T	exonic	ORF1a	.	nonsynonymous	ORF1a:cds-YP_	0.776	1.92395	0.826772	0.04	D	.	Peptidase_C16	.	.	.	.	.	orf1ab_SYTTTIK	-1	.	.	.	.	.	.	.	.	
1	6070	6070	C	T	exonic	ORF1a	.	synonymous SN	ORF1a:cds-YP_	-2.5	-0.408094	0.023622	0.13	T	.	.	.	.	.	orf1ab polyprotei	16	.	.	.	.	.	.	.	.	.	.	
1	6294	6294	T	C	exonic	ORF1a	.	nonsynonymous	ORF1a:cds-YP_	1.61	2.12403	1	0	D	.	.	.	.	.	.	.	orf1ab_TYKPNT	0.07	.	.	.	.	.	.	.	.	
1	6355	6355	A	G	exonic	ORF1a	.	synonymous SN	ORF1a:cds-YP_	1.61	2.23063	1	0.88	T	.	.	.	.	.	.	.	.	.	.	.	.	.	.	.	.	.	
1	9448	9448	C	T	exonic	ORF1a	.	synonymous SN	ORF1a:cds-YP_	-3.3	-0.224827	0.992134	0.14	T	.	.	.	Transmembrane	.	orf1ab polyprotei	17	.	.	.	.	.	.	.	.	.	.	
1	9707	9707	A	G	exonic	ORF1a	.	nonsynonymous	ORF1a:cds-YP_	1.65	2.23647	1	1	T	.	.	.	Transmembrane	.	orf1ab polyprotei	7.3	.	.	.	.	.	.	.	.	.	.	
1	9891	9891	C	T	exonic	ORF1a	.	nonsynonymous	ORF1a:cds-YP_	1.65	3.30935	1	0.01	D	.	.	.	.	.	.	.	orf1ab_TQYNRY	0.04	.	.	.	.	.	.	.	.	
1	9943	9943	C	T	exonic	ORF1a	.	synonymous SN	ORF1a:cds-YP_	-0.78	0.406276	1	1	T	.	.	.	.	.	.	.	orf1ab_GAMDTT	0.09	.	.	.	.	.	.	.	.	
1	11195	11195	C	T	exonic	ORF1a	.	nonsynonymous	ORF1a:cds-YP_	0.68	1.60466	1	0.03	D	.	.	.	Transmembrane	.	orf1ab polyprotei	18	orf1ab_FLLPSLA	-1	.	.	.	.	.	.	.	.	
1	11461	11461	C	T	exonic	ORF1a	.	synonymous SN	ORF1a:cds-YP_	0.756	1.8523	1	0.8	T	.	.	.	Transmembrane	.	orf1ab polyprotei	9.8	orf1ab_NALDQA	0.04	.	.	.	.	.	.	.	.	
1	11619	11619	T	C	exonic	ORF1a	.	nonsynonymous	ORF1a:cds-YP_	0.411	0.890819	1	0.25	T	.	.	.	Transmembrane	.	.	.	.	.	.	.	.	.	.	.	.	.	
1	11950	11950	C	T	exonic	ORF1a	.	synonymous SN	ORF1a:cds-YP_	-0.159	0.684787	1	1	T	.	.	.	.	.	.	.	.	.	.	.	.	.	.	.	.	.	
1	12017	12017	T	G	exonic	ORF1a	.	nonsynonymous	ORF1a:cds-YP_	-3.3	-0.208016	0.0745827	0	D	.	.	.	.	.	orf1ab polyprotei	18	.	.	.	.	.	.	.	.	.	.	
1	12325	12325	C	T	exonic	ORF1a	.	synonymous SN	ORF1a:cds-YP_	0.787	1.97211	1	1	T	.	.	.	.	.	.	.	.	.	.	.	.	.	.	.	.	.	
1	13085	13085	G	A	exonic	ORF1a	.	nonsynonymous	ORF1a:cds-YP_	1.65	4.256	1	0	D	.	.	.	.	.	orf1ab polyprotei	12	orf1ab_FAVDAA	-1	.	.	nCoV-2019_43_	.	.	.	.	.	
1	13414	13414	T	C	exonic	ORF1a	.	synonymous SN	ORF1a:cds-YP_	1.65	2.19337	1	1	T	.	.	.	.	.	.	.	.	.	.	.	.	.	.	.	.	.	
1	13957	13957	C	T	exonic	ORF1b	.	nonsynonymous	ORF1b:cds-YP_	1.65	3.31844	1	0.06	T	.	.	.	.	.	orf1ab polyprotei	15	orf1ab_FVENPD	-1	.	.	.	.	.	.	.	.	
1	14120	14120	C	T	exonic	ORF1b	.	nonsynonymous	ORF1b:cds-YP_	-0.127	1.19331	1	0	D	.	.	.	.	.	.	.	.	.	.	.	.	SARS-CoV-2_IB	Korea	.	.	.	
1	14857	14857	G	T	exonic	ORF1b	.	nonsynonymous	ORF1b:cds-YP_	0.741	2.74953	1	0.06	T	.	.	.	.	.	.	.	orf1ab_FVVEVVI	0.06	.	.	.	.	.	.	.	.	
1	14874	14874	G	T	exonic	ORF1b	.	nonsynonymous	ORF1b:cds-YP_	-3.3	0.0713543	0.992646	0.01	D	.	.	.	.	.	.	.	orf1ab_FVVEVVI	0.06	.	.	nCoV-2019_50_	.	.	.	.	.	
1	15546	15546	C	A	exonic	ORF1b	.	synonymous SN	ORF1b:cds-YP_	-3.3	-0.805717	0.945323	1	T	.	RdRp_catalytic	.	.	.	.	.	.	.	.	.	nCoV-2019_51_	SARS-CoV-2_IB	Korea	.	.	.	
1	16188	16188	G	T	exonic	ORF1b	.	nonsynonymous	ORF1b:cds-YP_	1.65	4.256	1	0.03	D	.	.	.	.	.	.	.	.	.	.	.	nCoV-2019_53_	.	.	.	.	.	
1	16402	16402	G	T	exonic	ORF1b	.	nonsynonymous	ORF1b:cds-YP_	1.65	4.256	1	0.02	D	.	CV_ZBD	.	.	.	.	.	orf1ab_DVTDVT	-1	.	.	.	.	.	.	.	.	
1	16557	16557	T	C	exonic	ORF1b	.	synonymous SN	ORF1b:cds-YP_	-0.78	0.324472	1	1	T	.	.	.	.	.	.	.	.	.	.	.	.	.	.	.	.	.	
1	17462	17462	G	A	exonic	ORF1b	.	nonsynonymous	ORF1b:cds-YP_	1.65	4.256	1	0	D	.	#ERROR!	.	.	.	.	.	orf1ab_APRTLLT	-1	.	.	.	.	.	.	.	.	
1	17470	17470	C	T	exonic	ORF1b	.	synonymous SN	ORF1b:cds-YP_	1.65	3.32576	1	1	T	.	#ERROR!	.	.	.	.	.	orf1ab_APRTLLT	-1	.	.	.	.	.	.	.	.	
1	17479	17479	G	A	exonic	ORF1b	.	nonsynonymous	ORF1b:cds-YP_	1.65	4.256	1	0	D	.	#ERROR!	.	.	.	.	.	orf1ab_APRTLLT	-1	.	.	.	.	.	.	.	.	
1	17808	17808	G	T	exonic	ORF1b	.	nonsynonymous	ORF1b:cds-YP_	-0.793	1.00017	1	0.01	D	.	#ERROR!	.	.	.	orf1ab polyprotei	17	orf1ab_SPYNSQ	0.11	.	.	.	.	.	.	.	.	
1	18008	18008	A	G	exonic	ORF1b	.	nonsynonymous	ORF1b:cds-YP_	1.65	2.24049	1	0.23	T	.	#ERROR!	.	.	.	.	.	.	.	.	.	.	.	.	.	.	.	
1	18246	18246	C	T	exonic	ORF1b	.	synonymous SN	ORF1b:cds-YP_	1.65	3.32576	1	1	T	.	.	.	.	.	.	.	.	.	.	.	.	.	.	.	.	.	
1	18496	18496	A	G	exonic	ORF1b	.	nonsynonymous	ORF1b:cds-YP_	1.65	2.21939	1	0.01	D	.	.	.	.	.	orf1ab polyprotei	19	.	.	.	.	.	.	.	.	.	.	
1	18647	18647	C	T	exonic	ORF1b	.	nonsynonymous	ORF1b:cds-YP_	1.65	3.27843	1	0.09	T	.	.	.	.	.	.	.	.	.	.	.	.	.	.	.	.	.	
1	19153	19153	A	G	exonic	ORF1b	.	nonsynonymous	ORF1b:cds-YP_	1.65	2.21939	1	0.78	T	.	.	.	.	.	orf1ab polyprotei	17	orf1ab_SYATHS	-1	.	.	.	.	.	.	.	.	
1	19224	19224	T	C	exonic	ORF1b	.	synonymous SN	ORF1b:cds-YP_	-0.794	0.427165	1	1	T	.	.	.	.	.	.	.	orf1ab_YPANSIV	0.08	.	.	nCoV-2019_64_	.	.	.	.	.	
1	20302	20302	G	A	exonic	ORF1b	.	nonsynonymous	ORF1b:cds-YP_	1.65	4.256	0.984252	0.1	T	.	.	.	.	.	.	.	orf1ab_RYKLEG	-1	.	.	.	.	.	.	.	.	
1	20413	20413	T	C	exonic	ORF1b	.	synonymous SN	ORF1b:cds-YP_	-3.3	-0.0514961	0.88189	0.49	T	.	.	.	.	.	orf1ab polyprotei	20	orf1ab_FELEDFI	0.08	.	.	.	.	.	.	.	.	
1	20569	20569	G	T	exonic	ORF1b	.	nonsynonymous	ORF1b:cds-YP_	1.65	4.256	1	0	D	.	.	.	.	.	orf1ab polyprotei	18	.	.	.	.	nCoV-2019_67_	.	.	.	.	.	
1	20578	20578	G	T	exonic	ORF1b	.	nonsynonymous	ORF1b:cds-YP_	1.65	4.256	1	0.01	D	.	.	.	.	.	.	.	.	.	.	.	.	.	.	.	.	.	
1	20703	20703	C	T	exonic	ORF1b	.	synonymous SN	ORF1b:cds-YP_	-3.3	-3.70061	0	1	T	.	.	.	.	.	.	.	orf1ab_GVAMPN	-1	.	.	.	.	.	.	.	.	
1	20940	20940	G	T	exonic	ORF1b	.	synonymous SN	ORF1b:cds-YP_	0.482	0.730921	1	0.38	T	.	.	.	.	.	.	.	.	.	.	.	.	.	.	.	.	.	
1	21008	21008	C	T	exonic	ORF1b	.	nonsynonymous	ORF1b:cds-YP_	1.65	3.34955	0.937008	0	D	.	.	.	.	.	.	.	orf1ab_TLIGDCA	0.09	.	.	.	.	.	.	.	.	
1	21017	21017	C	T	exonic	ORF1b	.	nonsynonymous	ORF1b:cds-YP_	1.65	3.34955	1	0.93	T	.	.	.	.	.	.	.	.	.	.	.	.	.	.	.	.	.	
1	21071	21071	A	G	exonic	ORF1b	.	nonsynonymous	ORF1b:cds-YP_	1.65	2.24167	1	0.61	T	.	.	.	.	.	.	.	.	.	.	.	.	.	.	.	.	.	
1	21077	21077	C	T	exonic	ORF1b	.	nonsynonymous	ORF1b:cds-YP_	-2.44	0.428772	0.440945	0.11	T	.	.	.	.	.	.	.	.	.	.	.	nCoV-2019_70_	.	.	.	.	.	
1	21486	21486	T	C	exonic	ORF1b	.	synonymous SN	ORF1b:cds-YP_	-3.3	-0.779827	0.023622	0.41	T	.	.	.	.	.	orf1ab polyprotei	11	.	.	.	.	.	.	.	.	.	.	
1	21621	21621	C	T	exonic	S	.	nonsynonymous	S:cds-YP_00972	-3.1	-5.19735	0	0.12	T	disulf_bond	BetaCoV_S1-NT	.	.	Surface glycopro	.	.	surface glycoprot	0.12	.	.	.	.	.	.	.	.	
1	21846	21846	C	T	exonic	S	.	nonsynonymous	S:cds-YP_00972	-0.152	0.679055	0	0.07	T	disulf_bond	BetaCoV_S1-NT	.	.	.	.	.	surface glycoprot	-1	.	.	.	.	.	.	.	.	
1	21933	21933	C	T	exonic	S	.	nonsynonymous	S:cds-YP_00972	-0.133	0.679055	0.00787402	0.41	T	disulf_bond	BetaCoV_S1-NT	.	.	Surface glycopro	surface glycoprot	14	.	.	.	.	.	.	.	.	.	.	
1	21952	21952	C	T	exonic	S	.	synonymous SN	S:cds-YP_00972	-3.3	-2.51465	0	1	T	disulf_bond	BetaCoV_S1-NT	.	.	.	surface glycoprot	14	.	.	.	.	.	.	.	.	.	.	
1	22032	22032	T	C	exonic	S	.	nonsynonymous	S:cds-YP_00972	-0.866	1.0623	0.00787402	0.52	T	disulf_bond	BetaCoV_S1-NT	.	.	Surface glycopro	surface glycoprot	18	.	.	.	.	nCoV-2019_72_	.	.	.	.	.	
1	22201	22201	G	T	exonic	S	.	synonymous SN	S:cds-YP_00972	-3.3	-4.17537	0	0.51	T	.	BetaCoV_S1-NT	.	.	.	.	.	surface glycoprot	0.08	.	.	.	.	.	.	.	.	
1	22448	22448	C	A	exonic	S	.	nonsynonymous	S:cds-YP_00972	-0.152	0.679055	0.00787402	0.29	T	disulf_bond	BetaCoV_S1-NT	.	.	.	.	.	.	.	.	.	.	.	.	.	.	.	
1	23719	23719	T	C	exonic	S	.	synonymous SN	S:cds-YP_00972	-3.3	-0.235276	0.944882	1	T	.	.	.	.	Surface glycopro	.	.	surface glycoprot	0.11	.	.	.	.	.	.	.	.	
1	24794	24794	G	A	exonic	S	.	nonsynonymous	S:cds-YP_00972	1.65	4.256	0.850394	0.35	T	.	.	.	.	.	.	.	.	.	.	.	.	.	.	.	.	.	
1	25169	25169	C	T	exonic	S	.	nonsynonymous	S:cds-YP_00972	-0.194	-0.113244	0.944882	0.01	D	.	.	.	.	.	.	.	surface glycoprot	-1	Spike glycoprote	KNHTSPDVDLG	.	.	.	.	.	.	
1	25281	25281	G	A	exonic	S	.	nonsynonymous	S:cds-YP_00972	1.65	4.256	1	0	D	.	.	.	.	.	.	.	.	.	.	.	nCoV-2019_84_	.	.	.	.	.	
1	25546	25546	C	T	exonic	ORF3a	.	nonsynonymous	ORF3a:cds-YP_	-3.3	-1.39205	0.685039	0.27	T	.	.	.	Transmembrane	.	ORF3a protein_A	11	.	.	.	.	.	.	.	.	.	.	
1	25703	25703	C	T	exonic	ORF3a	.	nonsynonymous	ORF3a:cds-YP_	0.597	0.612417	0.732283	0.23	T	.	.	.	Transmembrane	.	.	.	ORF3a protein_	-1	.	.	.	.	.	.	.	.	
1	25812	25812	T	C	exonic	ORF3a	.	synonymous SN	ORF3a:cds-YP_	-3.3	-3.77857	0.023622	1	T	.	.	.	.	.	ORF3a protein_K	13	ORF3a protein_N	0.03	.	.	.	.	.	.	.	.	
1	26113	26113	G	T	exonic	ORF3a	.	stopgain	ORF3a:cds-YP_	0.154	0.238717	0	.	.	.	.	.	.	.	.	.	ORF3a protein_E	-1	.	.	.	.	.	.	.	.	
1	26367	26367	G	C	exonic	E	.	synonymous SN	E:cds-YP_00972	0.715	2.57435	1	1	T	.	Intravirion	.	.	.	envelope protein_	15	.	.	.	.	.	SARS-CoV-2_IB	Korea	.	.	.	
1	26486	26486	T	A	upstream;downst	M;ORF6;ORF7a;	dist=37;dist=14	.	.	1.65	2.20065	1	.	.	.	.	.	.	.	.	.	.	.	.	.	.	.	.	.	.	.	
1	26933	26933	A	G	exonic	M	.	synonymous SN	M:cds-YP_0097	1.65	2.20759	1	1	T	.	Intravirion	.	.	.	membrane glyco	20	membrane glyco	0.08	.	.	.	.	.	.	.	.	
1	27643	27643	C	A	exonic	ORF7a	.	nonsynonymous	ORF7a:cds-YP_	1.65	3.2857	1	0	D	.	.	.	.	.	ORF7a protein_V	15	ORF7a protein_F	0.1	.	.	.	.	.	.	.	.	
1	28024	28024	A	G	exonic	ORF8	.	nonsynonymous	ORF8:cds-YP_0	-0.11	0.731118	0	0.16	T	.	.	.	.	.	ORF8 protein_FY	13	.	.	.	.	.	.	.	.	.	.	
1	28028	28028	G	A	exonic	ORF8	.	stopgain	ORF8:cds-YP_0	0.372	1.22069	0.165354	.	.	.	.	.	.	.	ORF8 protein_FY	13	.	.	.	.	.	.	.	.	.	.	
1	29659	29659	C	T	exonic	ORF10	.	synonymous SN	ORF10:cds-YP_	1.6	3.25353	1	.	.	.	.	.	.	.	.	.	.	.	.	.	.	.	.	.	.	.	
