# Supplementary material for: Initial Insights Into the Genetic Epidemiology of SARS-CoV-2 Isolates From Kerala Suggest Local Spread From Limited Introductions
Source: Front Genet. 2021 Mar 17;12:630542. doi: 10.3389/fgene.2021.630542 (PMC8010186; doi:10.3389/fgene.2021.630542)
Supplement: Supplementary Table 12A — Tabulation of read alignment statistics for variants spanning Primer/Probe sites. [file Data_Sheet_8.PDF]

Variant	Occurrence	Frequency	Total Read Count	Reference Base Count	Alternate Base Count	Percentage of variation
**14120C>T**	1	0.56%	13478	C - 5	T - 13472	100%
			2398	C - 0	T - 2398	100%
			880	C - 0	T - 880	100%
			444	C - 0	T - 444	100%
			11	C - 0	T - 11	100%
			23	C - 0	T - 23	100%
			853	C - 0	T - 853	100%
			3646	C - 0	T - 3646	100%
**22444C>T**	8	4.469%)	115	C - 0	T - 115	100%
			14272	G - 0	C - 14272	100%
**26367G>C**	2	1.12%	7553	G - 2	C - 7551	100%
			5502	C - 1	T - 5501	100%
			3218	C - 0	T - 3218	100%
			2917	C - 0	T - 2917	100%
			218	C - 0	T - 218	100%
			164	C - 0	T - 164	100%
**28854C>T**	6	3.35%	1909	C - 0	T - 1909	100%
			171	G - 0	T - 171	100%
**28899G>T**	2	1.12%	2513	G - 8	T - 2505	100%
